# Impacts of Global School Feeding Programmes on Children’s Health and Wellbeing Outcomes: A Scoping Review

**DOI:** 10.1136/bmjopen-2024-093244

**Published:** 2025-10-02

**Authors:** Amy Locke, M James, Hope Jones, Rachel Davies, Francesca Williams, Sinead Brophy

**Affiliations:** 1 Centre for Population Health, Swansea University, Swansea, UK

**Keywords:** Health, Schools, Nutrition, PUBLIC HEALTH

## Abstract

**Abstract:**

**Objectives:**

School feeding programmes (SFPs) are widely implemented to address child poverty, food insecurity and malnutrition, yet evidence on their influence on children’s health outcomes is limited. With ongoing debate around universal versus targeted provision, this scoping review aims to map global literature on SFPs, identify which health and well-being outcomes are reported, and explore how these outcomes vary by programme type (targeted vs universal).

**Design:**

Scoping review conducted in accordance with the Preferred Reporting Items for Systematic Reviews and Meta-Analyses guidelines. The protocol was pre-registered on the Open Science Framework.

**Data sources:**

Four electronic databases—Medline, PubMed, Web of Science and Google Scholar—were searched in December 2023 and July 2025. Reference lists of included papers were also screened.

**Eligibility criteria:**

Included studies examined the impact of SFPs on physical, emotional, psychological and social health outcomes in children aged 5–16. Only English-language studies published between 2009 and 2025 were included.

**Data extraction and synthesis:**

Data were extracted using a structured template and reviewed by multiple authors. Due to the heterogeneity in study designs and reported outcomes, a narrative synthesis approach was used to group findings thematically, following established guidance for narrative synthesis in systematic reviews.

**Results:**

A total of 44 papers were included in the final review, spanning 13 countries and published between 2009 and 2025. SFPs were associated with healthier weight status, improved dietary intake, better social engagement and reductions in stigma. Targeted programmes addressed food insecurity but were more often linked to stigma and poorer mental health outcomes.

**Conclusions:**

Universal SFP were effective at improving children’s health outcomes such as healthy weight, improved behaviour and social support. Overall, both targeted and universal SFP positively impact children’s health outcomes and address health disparities.

**STRENGTHS AND LIMITATIONS OF THIS STUDY**

- The review mapped both universal and targeted provisions, providing a broad overview of the existing literature.
- The review included an appraisal of the methodological quality of the included studies.
- The review only included studies in English.
- Studies which explored the impacts on academic attainment were excluded.

## Introduction

 School feeding programmes (SFPs) are among the most widely implemented interventions intended to support child nutrition, improve food security and reduce some of the immediate effects of poverty on children’s well-being.^1^ In 2022, 418 million children worldwide received a free or subsidised school meal as part of a SFP.^2^ In recent years, growing concerns have arisen regarding the nutritional health of children.^3^ Schools present an opportunity to promote healthy eating habits among children and can serve as an integrated platform for delivering nutrition interventions, as highlighted in the global statement on school-based food and nutrition systems by Hunter *et al*.^4^ Schools can also serve as a preventative measure to negative health implications.^5^ From a public health perspective, schools are ideal settings to promote healthy eating behaviours early in life since children have access to at least one main meal per day at school in most schools.^6^ There is a large body of literature dedicated to exploring the impacts of SFP on educational outcomes, noting positive outcomes, specifically in relation to key stage attainment and attendance.^7 8^ However, literature relating to the impacts of SFP on children’s health outcomes is limited, and even less research considers SFP as a preventative tool for public health. In this review, children’s health outcomes are grouped into four domains: physical (eg, diet, weight), psychological, emotional (eg, well-being, behaviour) and social (eg, peer relationships, inclusion). Research by Chaudhary *et al*^9^ demonstrated that initiatives targeting food and nutrition within schools could enhance dietary habits, promote healthy eating and impact body measurements positively. Noting that interventions with a focus on meal provision, healthy eating promotion and food literacy are most effective in improving children’s health outcomes. It is well documented that good nutrition plays a vital role in maintaining good health throughout the life course, preventing malnutrition in all forms and decreasing the risk of non-communicable diseases, such as cardiovascular disease, diabetes and some cancers.^10^ Increases in processed foods and changes in lifestyles have led to a shift in dietary patterns, with individuals opting for cheaper food options and convenience foods.^11^ With the rise in poor dietary habits, there is a negative impact on health outcomes. This has become a public health concern, specifically in relation to rising childhood obesity. Figures from 2022 indicate childhood obesity has reached an all-time high with an estimated 390 million children and adolescents aged 5–19 years being overweight or living with obesity. Of this, 37 million children under age 5 were classified as overweight.^12 13^ To date, no country is on track to curb the obesity crisis.^14^ Moreover, in 2019, over 161 million children under the age of 5 were affected by undernutrition globally.^15^ Nutritional deficiencies associated with undernutrition have been linked to childhood wasting, stunting, infections, and cognitive and behavioural disorders.^16^,^18^

Health disparities due to poor nutritional intake are largely seen in poor economic groups and often arise in early childhood, resulting in ongoing implications into adulthood.^19^ According to the WHO,^5^ childhood and adolescence are critical periods for promoting nutritional health and reducing the risk of negative health outcomes. Calls for policy reform to SFPs to improve children’s diet quality are increasingly common in public health literature.

Despite the large potential health benefits of SFP, they have proven difficult to evaluate given the diversity in implementation policies across nations. For example, Europe alone has a range of SFP policies from universal to targeted approaches.^20^ Universal systems refer to a provision open to all students, regardless of socioeconomic status.^21^ This system is well established in countries such as Sweden, where all children in both primary and secondary education (ages 5–16) have access to at least one free school meal (FSM) a day and is regarded as a symbol of national welfare.^22^ However, there is also diversity among policies relating to universal systems, whereby countries offer universal school meals to children in particular year groups. For instance, Latvia offers free meals to children in grades 1 to 4 (ages 7–9), and Lithuania provides FSM from preschool to first grade (up to age 7).^20^

Targeted programmes, on the other hand, target children from lower socioeconomic backgrounds, usually using a means-tested eligibility system which is based on parental income.^23^ This approach is implemented in Poland,^24^ Slovenia^25^ and parts of the UK.^26^ This approach, while beneficial to some families, has its own limitations. For example, a threshold cut-off means families slightly above the eligibility criteria may still be living in poverty but are able to access FSM, and the stigma associated with being eligible may lead to a lack of uptake.^21^ There is considerable debate among government policy makers as to whether SFP should be targeted (benefit eligibility depends on family income) or universal (benefits are provided to all students with no eligibility criteria).^27 28^ However, given the notable gap in health-related literature, it is difficult to establish which system, if any, is most effective at addressing health disparities.

To our knowledge, no study has systematically mapped or synthesised the existing literature on how SFPs are reported to influence children’s health outcomes. Therefore, this review aims to address the following questions: (1) To what extent does the existing literature examine the impacts of SFPs on the physical, emotional, psychological and social health of school children globally; (2) What challenges, including factors influencing uptake, are reported in the literature regarding the implementation of FSM provision; and (3) How do reported health outcomes associated with SFP differ between targeted and universal provision models?

## Methods

### Review design

We conducted a scoping review of qualitative and quantitative research to map current evidence on the reported impacts of SFPs on children’s health outcomes globally. This scoping review was conducted in accordance with the Preferred Reporting Items for Systematic Reviews and Meta-Analysis (PRISMA) guidelines.^29^ The protocol for the review was registered on the Open Science Framework.^30^ A preliminary search of MEDLINE and the Cochrane Database of Systematic Reviews was conducted, and no current or underway systematic reviews or scoping reviews on the topic were identified. The study findings were summarised through a narrative synthesis. Researcher (AL) extracted study data from full-text papers. Each extraction was reviewed by a second reviewer. Any conflicts were presented to and resolved through group discussion.

### Search strategy

Four electronic databases—Medline, PubMed, Web of Science and Google Scholar (including Science Direct, Web of Science, ProQuest Central, EBSCO, CINAHL)—were searched to identify relevant research papers in English. Only studies published in English were included due to resource limitations for translation and screening of non-English texts. While this may exclude some relevant international studies, it ensured feasibility and consistency in quality assessment and data extraction. Studies published from 2009 were included, with most papers from 2020 to 2023. This cut-off was selected to reflect recent developments in school feeding policies and global nutrition initiatives. The literature search was conducted by lead author (AL) in December 2023 with screening taking place in January and February 2024. Additionally, the reference lists of all included sources were also screened for additional studies. Reports and case studies were also sourced and included in the final screening. Search terms included the following: Universal Free School Meals OR School Feeding Programmes OR Free School Meals and Children’s Health OR Free School Meals and Children’s Wellbeing OR School Feeding Programmes OR Challenges with School Feeding Programmes OR School Meal Provision. Additionally, each string was searched using the phrase ‘Impacts of’. The complete search strategy for all databases is provided in online supplemental material S1.

### Eligibility

In line with the Joanna Briggs Institute (JBI) methodology for scoping reviews, this study used the Population–Concept–Context framework^31^ to guide the development of the eligibility criteria. The population of interest was school-aged children (5–16 years); the concept focused on SFPs (universal or targeted) and their reported impacts on health and well-being; and the context included global studies across all settings published in English between 2009 and 2023.

#### Inclusion criteria

Studies exploring SFPs were included if they met the following outcomes: (1) children’s physical health—illness, physical activity and/or dietary intake; (2) children’s emotional and psychological health—emotion regulation, resilience, self-esteem and behaviour; (3) children’s social health—social engagement/participation, building and maintaining relationships; (4) studies published within the past 15 years to ensure research is up to date and relevant; (5) primary and secondary school aged children (ages 5 to 16); and (6) studies investigating the implementation of universal feeding programmes and its potential challenges. Both experimental and quasi-experimental study designs included randomised controlled trials, non-randomised controlled trials, before and after studies, and interrupted time series studies. Descriptive observational study designs, including case series, individual case reports and descriptive cross-sectional studies, were also included. Qualitative studies were also included that focused on children’s self-assessed health and well-being and perceptions of SFPs.

#### Exclusion criteria

Studies were excluded if they met the following criteria: (1) children below or above school age (below age 5 or above 18); (2) studies with a focus on SFP and academic outcomes—performance, attainment or attendance without a health element; (3) studies that only looked at parental funded meals/packed lunches; (4) studies not in English; and (5) studies published prior to 2009. Additionally, any studies that did not focus on the impacts of school meal provisions on children’s health, unless considering factors influencing uptake, were excluded.

### Evidence selection

Selected papers were uploaded to Covidence systematic review software^32^ by the lead author (AL). After removing 30 duplicates, the remaining titles and abstracts (177) were screened against the selection criteria by authors (AL, MJ, HJ, RD, SB). Remaining studies undertook full-text screening by reviewers (AL, MJ, HJ, RD, FW). Any conflicts during the review process were resolved through group discussion. One paper was removed during data extraction as it became apparent that it was an abstract for a conference presentation and no full text was available. To ensure the review included the most recent evidence, an updated search was conducted on 7 July 2025 to capture any new publications from January 2024 to July 2025. The search and screening were conducted independently by AL and HJ using the original eligibility criteria. Of the 11 records identified, 2 met the inclusion criteria and were incorporated into the final review.

### Quality assessment

Methodological quality of included studies was assessed using the Mixed Methods Appraisal Tool (MMAT).^33^ The MMAT is a quality appraisal tool developed to evaluate the methodological quality of empirical studies. The MMAT was first published in 2009. Since then, it has been validated in several studies testing its inter-rater reliability, usability and content validity.^34^ The tool can appraise five different categories of study designs: qualitative, randomised controlled trial, non-randomised, quantitative descriptive and mixed methods studies. As our review includes numerous study designs, this tool was deemed appropriate. Each section of the papers is assessed based on five criteria for methodological quality, offering three responses: ‘Yes’ (indicating the criterion is satisfied), ‘No’ (indicating the criterion is not satisfied) and ‘Can’t tell’ (suggesting insufficient information to assess). Studies are categorised as low quality if they receive a ‘Yes’ response for two or less questions, moderate quality for three questions and high quality for four or more questions. Four papers included in the review were impact reports and opinion pieces and did not fit under an appraisal category within the MMAT; therefore, the modified version of the JBI critical appraisal checklist for text and opinion was used to quality assess these papers.^35^ Appraisal is subjected to the same response criteria as above; ‘Yes’ (indicating the criterion is satisfied), ‘No’ (indicating the criterion is not satisfied) and ‘Can’t tell’ (suggesting insufficient information to assess). No papers were excluded based on the results of the quality assessment. Quality assessment was conducted by one reviewer (AL) and checked by a second reviewer(s).

### Data extraction and analysis

Relevant information from selected studies was extracted using a template (this is provided in online supplemental information S2) developed by lead author (AL). The data collection template included information on author(s), year of publication, country, type of meal provision, participants, study design, and key findings, gaps/limitations identified and recommendations for future research. The development of the template was guided by the JBI manual for evidence synthesis^36^ and piloted on Covidence^32^ using two papers. Data analysis consisted of a narrative synthesis due to heterogeneity of both study designs and outcomes among included studies, making statistical meta-analysis impractical. Analysis and synthesis were conducted following recommendations from the Guidance of the Conduct of Narrative Synthesis in Systematic Reviews.^37^ Specifically, a grouping strategy was used to arrange findings into similar theme groups.

### Patient and public involvement

No patient and public involvement was used in this review.

## Results

### Overview of studies

**Figure 1:**
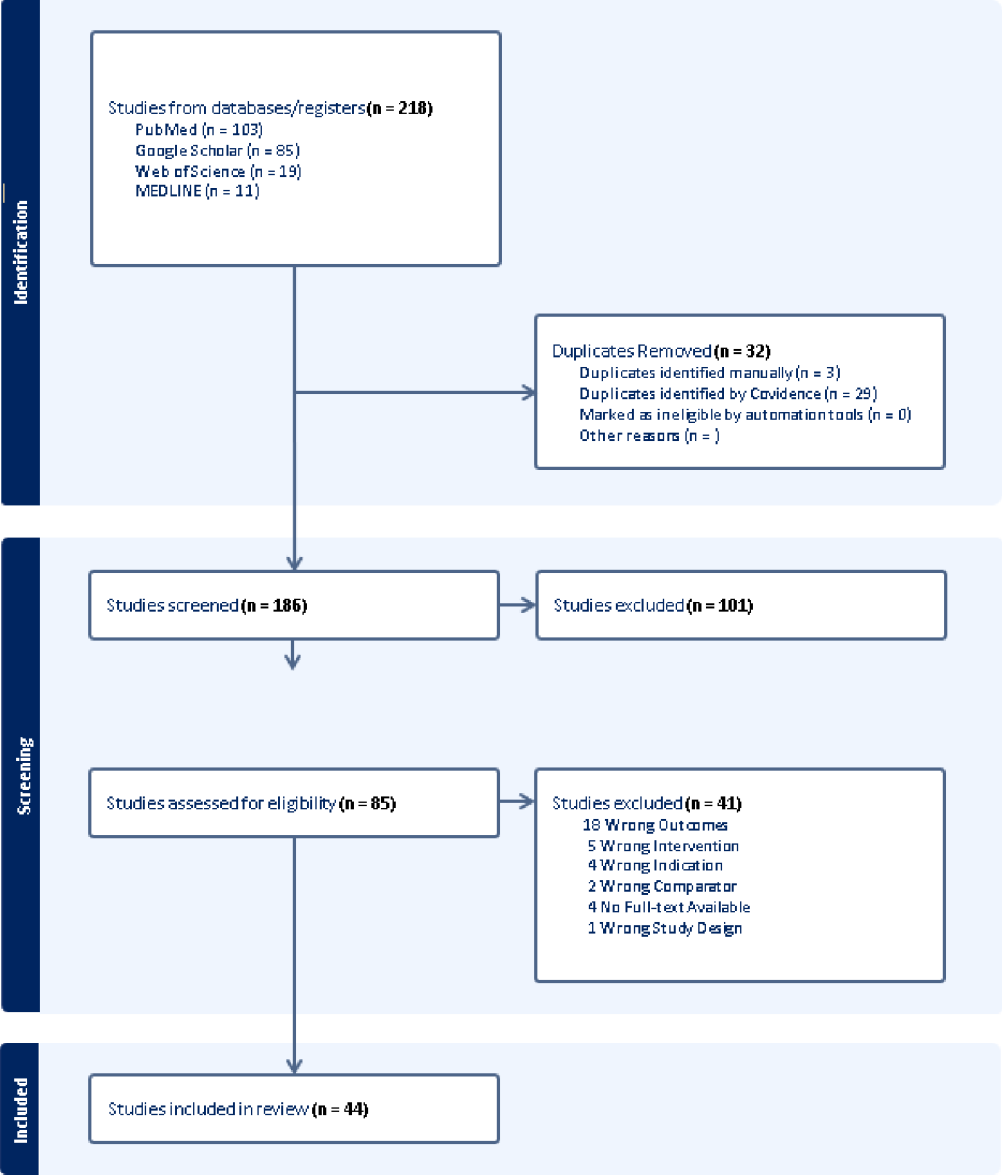
. PRISMA diagram. PRISMA, Preferred Reporting Items for Systematic Reviews and Meta-Analysis.

The literature search yielded a total of 218 titles, and these were imported to Covidence for title and abstract screening. After duplicates were removed (n=32), 186 titles and abstracts remained for screening. Of these, 101 were excluded for not meeting the inclusion criteria, that is, wrong outcomes measured. A further 41 studies did not meet the eligibility criteria and were excluded at full-text screening phase, with a majority vote for wrong outcomes studied. A total of 44 papers were included in the final review. A full summary of study characteristics and health-related findings is presented in supplementary information (online supplemental table S3). Figure 1 illustrates the screening process.

#### Quality of included papers

The quality assessment scores of included studies are shown in table 1. Studies assessed with MMAT^33^ were categorised as high overall. 28 studies were categorised as having high methodological quality, 8 were classed as moderate and 8 were classed as being of low quality. Details of each study’s quality rating and design are provided in table 1.

**Table 1:** : Characteristics of the included studies

Author	Country	Provision type	Participant school year	Design	Quality assessment
Persson and Fjellström^22^	Sweden	Universal	All	Article piece	High
Guio^20^	Europe	Other	N/A	Policy recommendation	High
Harper and Wood^38^	UK wide	Targeted	Primary	Report	High
Oostindjer *et al*^39^	Cross-national	Other	N/A	Discussion/essay	High
Kitchen *et al*^40^	England	Universal	All	Impact report	Moderate
Lundborg *et al*^19^	Sweden	Universal	Birth to 50+	Difference-in-difference	High
Bethmann and Cho^41^	South Korea	Universal	Aged 9–16	Difference-in-difference	High
Holford and Rabe^42^	England	Universal	Primary	Difference-in-difference	Low
Holford and Rabe^43^	England	Universal	Primary	Difference-in-difference	High
Parnham *et al*^44^	England and Scotland	Universal	Primary	Difference-in-difference	High
Vik *et al*^6^	Norway	Targeted	Secondary	Non-randomised control trial	High
Vik *et al*^45^	Norway	Universal	Secondary	Non-randomised control trial	High
Neervoort *et al*^46^	Kenya	Universal	Primary	Non-randomised control trial	Low
Meier *et al*^47^	USA	Targeted	N/A	Quantitative exploratory	Low
Batista *et al*^48^	Brazil	Targeted	Aged 4–14	Cross-sectional	Moderate
Colombo *et al*^49^	Sweden	Universal	Secondary	Cross-sectional	High
Horta *et al*^50^	Brazil	Targeted	Primary	Cross-sectional—analytical	High
James^51^	England	Targeted	Secondary	Cross-sectional—descriptive	Low
Long *et al*^52^	USA	Universal	All	Cross-sectional	High
Spence *et al*^53^	England	Universal (infant)	Primary	Cross-sectional	Moderate
Zailani *et al*^54^	Nigeria	Targeted	Primary	Cross-sectional	High
Evans^55^	England	Other	Primary	Cross-sectional—observational	High
Yang *et al*^56^	UK	Targeted	All	Cross-sectional	High
Davis *et al*^57^	Georgia— USA	Universal	All	Descriptive	Low
Altindag *et al*^58^	South Korea	Universal	All	Empirical analysis	Moderate
Goodchild *et al*^59^	England	Universal	Primary	Cross-sectional—descriptive	Moderate
Parnham *et al*^60^	UK	Targeted	All	Cross-sectional	Low
Zuercher *et al*^61^	USA	Universal	All	Cross-sectional	High
Jessiman *et al*^62^	England	Universal	Secondary	Mixed Methods	High
Hecht^64^	USA	Universal	All	Mixed methods	High
Taylor *et al*^65^	Vermont, USA	Universal	Staff	Mixed methods	Moderate
Yamaguchi *et al*^66^	Japan	Universal	Primary	Mixed methods	High
Cardoso *et al*^67^	Portugal	Targeted	Secondary	Qualitative	High
Goel *et al*^68^	USA—Virginia	Other	Primary	Observational	Moderate
Illøkken *et al*^69^	Norway	Targeted	Secondary	Qualitative	Low
McKelvie-Sebileau *et al*^70^	New Zealand	Universal	Secondary	Qualitative	High
Sahota *et al*^71^	England	Targeted	All	Qualitative	Moderate
Chelius *et al*^72^	USA	Universal	All	Qualitative	High
Mauer *et al*^73^	Norway	Targeted	Secondary	Qualitative	High
Carlisle *et al*^63^	England	Universal	Not specified	Mixed methods	High
Rahim *et al*^74^	England	Universal and targeted	Primary	Government report	High
Garton *et al*^75^	New Zealand	Universal	Not specified	Rapid narrative review	High
Chambers *et al*^76^	Scotland	Universal	N/A	Qualitative case study	Low
Chambers *et al*^77^	Scotland	Targeted	Secondary	Case study	High

N/A, Not Available (participant school year not reported).

Using the modified version of the JBI critical appraisal checklist for text and opinion,^35^ three papers were categorised as high, and the other was categorised as moderate. The four papers categorised as high^20 22 38 39^ used a variety of methodologies or a strong evidence base to support their findings/opinion. The paper rated moderate^40^ included less peer-reviewed evidence to support their findings, but methodological quality was satisfactory.

#### Characteristics of included studies

Characteristics of the included studies are summarised in table 1. Of the included studies, 5 used a difference-in-difference design,^1941^,^44^ 3 were non-randomised control trials,^6 45 46^ 15 were quantitative descriptive studies,^47^,^63^ 5 were mixed-methods^6264^,^66^ and 7 were qualitative.^67^,^73^ In addition, six were reports and/or opinion pieces,^2024 38^,^40 74^ one was a rapid narrative review^75^ and two were case studies.^76 77^

25 papers were based on universal SFPs.^1922 40^,^46 49 52 53 57^ 14 had targeted SFP systems.^6 38 47 48 50 51 54 56 60 67 69 71 73 77^ Four papers^20 39 55 68^ were categorised as ‘other’. One paper compared both targeted and universal SFPs.^74^ 11 papers included children across all school ages (primary and secondary) or used a broad age group described as ‘all’.^5 8 22 40 52 56 57 60 61 64 71 72^ 13 papers focused specifically on primary school children.^3842^,^44 46 50 53^ 11 papers were set in secondary school settings.^6 45 49 51 62 67 69 70 73 77^ Two papers spanned overlapping childhood age ranges that covered both primary and lower secondary (eg, aged 4–14^48^ and 9–16^41^). One study examined the impacts of SFPs across the life course, from birth to over age 50.^19^ One paper focused on school staff.^65^ Five studies did not explicitly state participant age or school level.^20 39 47 63 75 76^

**Figure 2:**
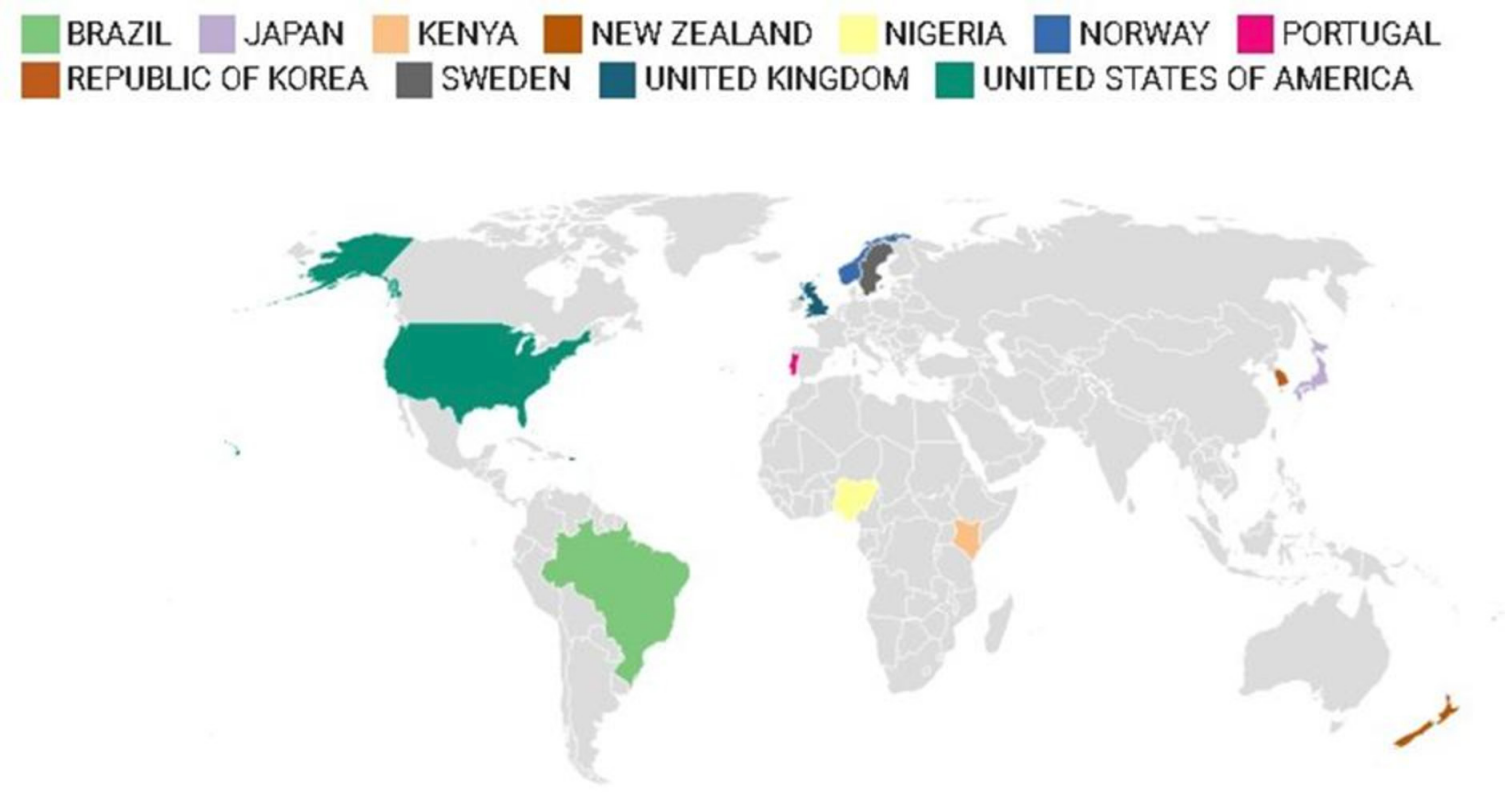
. Map showing countries included in review.

17 papers were from the UK (England n=11,^40 42 43 51 53 55 59 62 63 71 74^ Scotland n=2,^76 77^ England and Scotland n=1,^44^ UK wide n=3^38 56 60^). Eight papers were from the USA,^47 52 57 61 64 65 68 72^ three papers were from Sweden,^19 22 49^ four papers were from Norway,^6 45 69 73^ one paper was from Portugal^67^ and one paper was Europe wide.^20^ Two were from New Zealand,^70 75^ two were from Brazil,^48 50^ two were from South Korea,^41 58^ two were from Africa (Kenya^46^ and Nigeria^54^), one was from Japan^66^ and one was cross-national.^39^ Figure 2 demonstrates the countries of the included papers.

Overall studies that investigated universal feeding programmes appear to have the most significant positive outcomes on children’s health, specifically in relation to behaviour, health and socioeconomic outcomes. While targeted systems help to address food insecurity and child hunger, children eligible for targeted provision had a higher probability of experiencing poor mental health and stigma. Findings are discussed under the following themes/headings: *Physical Health: BMI and Body Weight Outcomes*, *Nutritional Intake*, *Psychological and Emotional Health Outcomes*, *Social Health Outcomes*, *Reducing Food Insecurity and Child Hunger*, *Factors Influencing Uptake* and *Wider Impacts*. These are described in more detail below.

##### Physical health: BMI and body weight outcomes

Five papers investigated the impacts of SFP on children’s body mass index (BMI) and weight outcomes. Two with targeted provision^6 48^ and three with a universal system.^41 42 57^ Studies had contradicting results.

Batista *et al*^48^ investigated the prevention of overweight children in Brazil through targeted SFP. The study which used a cross-sectional design and included 7017 participants from 21 schools found that being overweight was prevalent in 30.6% of children and being underweight was present in 1.9%. This study highlighted that a large proportion of the foods served were ultra-processed, recommending a re-evaluation of food standards within schools. Researchers noted an inability to observe extracurricular activities relating to food consumption and physical activity which may also influence children being overweight.

Bethman and Cho^41^ investigated the impacts of reintroducing universal free school meals (UFSM) in South Korea after the policy had been abolished a year prior. Findings indicated the removal of the policy had adverse effects on children’s BMI outcomes. The reintroduction of the policy on the other hand reversed these impacts and saw more children with healthier weights. However, the sample size of the study is not explicitly stated and may not represent the intended population.

Holford and Rabe^42^ suggested that longer exposure to a universal infant SFP positively influenced children’s weight outcomes. The study included 153 522 primary school children aged between 4 and 7 and used a difference-in-difference analysis to measure children’s weight outcomes over time. Findings concluded that children are more likely to be a healthy weight after exposure to the provision over the course of the first year of school. Though participants were very young, it is recommended that participants are tracked over a longer time period to see if this is a lasting effect. A study by Vik *et al*,^6^ conducted in a secondary school in Norway, investigated changes in children’s dietary habits, BMI and waist circumference modulated by socioeconomic status. The study conducted a non-randomised control trial consisting of two groups, non-exposure to SFP and exposure to SFP under a targeted system. Findings suggested FSMs increased the intake of healthy foods, particularly among children with lower socioeconomic status, leading to an increase in the Healthy Food score of the intervention group between baseline and follow-up. However, children in the intervention group had a significant increase in BMI, though it is not explicitly stated if the children were underweight prior to the trial. The study concluded that interventions to promote healthy eating are more effective in lower socioeconomic groups if they are free or at reduced price. Davis *et al*^57^ saw similar results when they examined the effects of a universal SFP in a deprivation area in the USA, reporting that BMI increased in provision schools versus non-provision schools. They further stated that children in provision schools had a higher BMI on average. In conclusion, there is no evidence that SFP lower BMI, but children are more likely to be a healthy weight. Factors such as the prevalence of ultra-processed foods, socioeconomic status and policy reintroduction played significant roles in these outcomes. Longer exposure to SFP, particularly universal programmes, was associated with healthier weight outcomes in younger children, though lasting effects require further study.

##### Nutritional intake

Ten papers^2244^,^46 49 53^ explored the impacts of SFP on nutritional intake. Outcomes explored were diverse. An article piece^22^ claimed that Sweden was one of the countries with the highest vegetable intake, with a substantial intake at lunch. Sweden has the longest running universal SFP in the world and supplies food to all school-aged children. The article notes that school meal provision in Sweden is seen as a universal welfare service and a part of public health work.

Vik *et al*^45^ used a non-randomised control trial including 164 secondary school children to investigate whether serving a free healthy school meal for 1 year resulted in a higher intake of fruit and vegetables and a lower intake of unhealthy snacks. Vik *et al*^45^ reported that a free healthy school meal for 1 year was associated with higher weekly intake of vegetables on sandwiches in the intervention group compared with the control group. However, it was not associated with a lower weekly intake of unhealthy snacks in the intervention group compared with the control group. It is important to note that the study had a small sample size and relied on self-reported data. Parnham *et al*^60^ examined the impact of a universal infant SFP policy on dietary intake in primary school children in England and Scotland. The implementation of the policy led to more children participating in school meals. No impact was seen on fruit and vegetable intake, but there was evidence that the policy lowered consumption of foods associated with packed lunches, such as crisps, and some nutrients, such as total fat and sodium. A further study by Evans^55^ also reported that school meals were a healthier option than packed lunches. The study used a cross-sectional design and included 2709 primary school children in England. Findings suggested children having a packed lunch consumed on average 11 g more total sugars than those who had a school meal, whereas children having a school meal were more likely to consume different types of vegetables and drink water. Overall, children with a packed lunch consumed a lower-quality diet over the whole school day. The study did not look directly at school meal provision and so it is difficult to note whether those on provision would yield differing results. Spence *et al*^53^ explored the effect of pre- and post-universal infant free school meals and school on pupil’s dietary intakes using a cross-sectional research design. At lunchtime, there was a statistically significant decrease in students’ non-milk extrinsic sugars intake pre-implementation and increases of intakes of cakes post-implementation. However, only two schools were included, thus limiting the generalisability of results. It is recommended that schools consider healthier policies when implementing SFP. Yamaguchi *et al*^66^ indicated that universal SFP are beneficial to individuals with low socioeconomic backgrounds. Their study, which used a mixed methods approach and included 719 primary school children in Japan, saw that children whose mothers were less educated had greater reliance on school lunch for their vegetable intake. Children with lower household income had more contribution from school lunch to their fruit intake. Household income was not explicitly considered in the study and mothers’ education was used to determine socioeconomic status, so it is unclear whether families were from lower socioeconomic backgrounds. Further studies are needed to conclude the correlation between vegetable intake and household income in Japanese children. Four papers, Neervoort *et al*,^46^ Zailani *et al*,^54^ Colombo *et al*^49^ and Goel,^68^ considered the impacts of SFP on vitamin intake as well as nutritional deficiencies. Neervoort *et al*^46^ conducted a non-randomised control trial including 67 primary school children to investigate the impacts of a universal SFP on anaemia, stunting, wasting and malnutrition. Findings indicated the programme reduced anaemia and malnutrition and improved child growth in the study group. While improvements were seen, the study had several limitations. Such as a very small sample size and participants were from only one school. The study also lacked information on the content and nutritional value of provided meals, which could influence programme effectiveness. Zailania *et al*^54^ investigated portion sizes of targeted school meals as well as nutrient intake and found that the meals served through the SFP contributed at least 33% for energy, protein, iron, calcium, sodium, vitamin A and zinc intake. However, there was no consumption of fruits, meat, poultry and fish within the programme. The study did not consider the contribution of other food sources, including snacks and homemade meals which may account for nutritional values. Colombo *et al*^49^ used a cross-sectional design to explore school lunch dietary intake under a universal SFP in Sweden. Results found a quarter of the overall energy intake; between 22% and 30% of selected nutrient intakes; almost half of vegetable intakes; roughly two-thirds of fish intakes; and around a third of red/processed meat intakes. These findings imply that school meals make an important contribution to children’s diets on weekdays. Researchers also reported school meals as more nutritious than meals consumed outside of school, though meals consumed outside of school were not measured. Goel *et al*^68^ examined total sugar in free breakfasts served in elementary (primary) schools in Virginia, USA, through an observational study. Findings suggest that meals offered might contribute to excessive overall sugar availability for children. Nutritional information for added sugars was not provided within the study; however, the findings may explain contradictions in BMI above. Goel *et al*^68^ state districts and policy makers should collaborate to implement more effective guidelines concerning sugar availability in breakfast items to optimise children’s dietary intake. In summary, the evidence for improved nutritional intake is limited. However, school meals are an important contribution to diets during weekdays. Consuming school meals alleviated dietary discrepancies related to social inequalities.

##### Psychological and emotional health outcomes

The impacts of SFP on children’s psychological and emotional outcomes were investigated in four papers.^41 56 58 65^ A randomised control trial by Altindag *et al*^58^ evaluated the effects of targeted school meals on students’ behaviour, bullying and violence in schools. Behavioural incidents have reduced by 35% since the implementation of a universal system in 2010 (particularly physical fights among students). It is believed that this reduction is the result of reduced stigma among students as socioeconomic status cannot be identified via the system. Provision did not impact students’ nutritional intake but reduced the stigma of receiving free meals through a targeted system by mitigating the possibility of identifying peers’ socioeconomic statuses. Taylor *et al*^65^ included 116 school staff in the USA to explore the impact of a universal SFP on school climate, behaviour and attainment. Taking a mixed-methods approach, over 725 of school staff surveyed reported that serving universal school meals has improved social climate as well as reductions in student stress, family financial stress and school administrator stress. This is thought to be a result of income differences being less visible, and the school community feeling more inclusive. It is recommended that further research include interviews with students and families to gain a better understanding. As previously mentioned, Bethman and Cho^41^ saw positive results when looking at children’s BMI. Similarly, the reintroduction of a universal policy also saw mental health improvements among students, proclaiming that free school lunches help to improve health and benefit student welfare. However, mental health was assessed using a ‘crying without any reason’ measure. The measure used is not reported so validity and reliability cannot be determined. No other mental health outcomes were assessed. A further paper by Yang *et al*^56^ assessed targeted school meals in the UK on children’s mental health outcomes. The study used a cross-sectional design and included 2166 primary and secondary children. Yang *et al*^56^ reported that poor mental health was observed in food insecure children receiving provision but also food insecure children not receiving provision. Interestingly, food insecure (ie, not having access to sufficient food) children receiving provision had a higher probability of poor mental health than those who were food insecure and not receiving provision. It is thought that this is because targeted approaches are accosted with stigma. In conclusion, universal approaches were associated with improved mental health outcomes, such as reduced behavioural incidents in school and an improved social climate. This was not observed with targeted approaches.

##### Social health outcomes

Five papers^62 63 69 71 73^ investigated the social impacts of SFP. The impacts of SFP are multifaceted with outcomes seen with social interactions, reduced stigma and narrowing inequalities. Findings are discussed further below. A mixed method, quasi-experimental evaluation exploring the impact of universal provisions in two London secondary schools by Carlisle *et al*^63^ saw improved social skills as children were eating with peers, improved behaviours, less stigma and students eating more varied foods. These findings were as a result of paired student interviews; this may have caused socially desirable responses. A further study by Jessiman *et al*^62^ which also included a sample of secondary school students in London also reported social health benefits. Students perceived feeling equal under a universal system, whereas they reported feeling ‘weak’ under the targeted system.

Illøkken *et al*^69^ conducted a qualitative study in a Norwegian secondary school, involving 13 students and five teachers to investigate the effects of a targeted school feeding programme on social inequalities. Participants viewed school meals as a social event where students made new friends and learnt new skills. It was also reported that social equality among students increased. However, it is unclear whether this was the result of the feeding programme or school meals in general. Another qualitative study conducted in a Norwegian secondary school by Mauer *et al*^73^ also reported positive social outcomes. Students expressed that social time while eating school meals was important to them. This study also noted that the popularity of the food was also important for attracting students to school meals. Sahota *et al*^71^ investigated factors influencing uptake of targeted school meals through focus groups with children in both primary and secondary schools in England, using focus groups to gain children’s perspectives. Food choice, queuing and the social aspects of lunch time, such as eating with friends, were a major influence in uptake. Recognition of the importance of the social aspects of dining for pupils and facilitation of social interactions through the spatial (including flexible locations, for example, outside) and temporal organisation of lunchtimes. It is reported that the schools involved in the study had a high level of targeted school meal entitlement which may have resulted in the normalisation of school meal uptake. It is recommended that social aspects of school meal provision be further investigated in schools with lower levels of eligibility to see if results differ.

##### Reducing food insecurity and child hunger

Three papers^50 64 75^ looked at the impacts of SFP on food insecurity and child hunger. Most papers in this review touched on food insecurity; however, these three papers specifically looked at impacts of food insecurity outcome. A rapid narrative review by Garton *et al*^75^ concluded that SFP in New Zealand significantly reduced hunger and food insecurity in primary schools. Hecht^64^ suggested that access to free meals through a universal provision in a high deprivation area in the USA reduced food insecurity among children as well as decreased child hunger. Last, a cross-sectional analysis by Horta^50^ investigated impacts of SFP on vulnerability risk. Findings saw positive impacts from consuming school meals on children’s diets, particularly among children living in high/very high social vulnerability risk areas.

##### Factors influencing uptake

10 papers explicitly investigated SFP participation and factors associated with non-take-up among students. Two studies gained perspectives through qualitative methods.^67 70^ A paper by McKelvie-Sebileau *et al*,^70^ involving universal provision, found that lack of knowledge of the programme and loss of agency over meal choices were major drivers of non-take-up, but positive influences such as participants’ food security, better nutritional knowledge and improved well-being were also perceived. Cardosa *et al*^67^ who evaluated a targeted approach in Portugal noted that quality of food was a concern when participating in school meals. Children who were not entitled to free or discounted meals reported eating at school less often than children with free or discounted lunches.

Three reports, Kitchen *et al*,^40^ Rahim *et al*^74^ and Harper and Wood,^38^ all reported increased take-up under a universal policy, for both previously non-eligible children and eligible children. It is suggested that improvements were seen as a result of reduced stigma and familiarising parents with school meals. Participants believed the pilot increased the range of food that pupils would eat, built their social skills at mealtimes and, for some pupils, resulted in health benefits associated with having a balanced meal, such as more energy, concentration and alertness and improved complexion. Uptake was higher among primary school children than secondary school children. However, Rahim *et al*^74^ in particular found schools experienced difficulty predicting the appropriate quantities of food required for each menu option, which meant that some meals either ran out early or were wasted. A study by Holford and Rabe^42^ also found that take-up of school meals in England by non-eligible children rose from a consistent 30–35% in the 8 years preceding the policy to approximately 85% in the universal period.

Additionally, two case studies,^76 77^ one exploring a targeted system and the other a universal system, saw similar results. Under the targeted provision^76^ stigma, food quality and social aspects played a vital role in take-up. Results specifically noted that being separated from friends decreased take-up. Implementation of a universal system,^77^ however, yielded more positive results such as families who were previously above the eligibility threshold now being able to access provision. It was also documented that school-provided meals were of a higher nutritional quality than those from home, exposing children to a greater variety of foods and establishing healthier eating habits. Finally, a cross-national comparison by Oostindjer *et al*^39^ indicated that removing popular foods such as meat from school menus significantly reduced take-up of school meals. This was particularly evident in Finland where meat and fish were removed from the school menu on some days reducing participation rate, consequently producing up to 60% plate waste. Please note that this paper did not explicitly mention SFP, but instead school menu choices and take-up; therefore, more research is needed on food choices under SFP to evaluate if there is an effect on take-up.

Our recent search found two additional papers^61 72^ with a focus on meal participation under universal SFPs. Zuercher *et al*.^61^ found that student participation in school meals increased significantly under universal provision. The study which included an online qualitative survey for school staff noted that 30.9% of participants reported decreased stigma and a 64.2% increase in uptake. It should be noted, however, that this study did not include students or families and recommends future research includes student experiences. A more recent study by Chelius *et al*,^72^ however, did explore student experiences. Findings indicated that the provision normalised participation in school lunch, thus reducing stigma among peers and leading to greater participation.

##### Wider impacts

The remaining seven papers did not fit into a thematic category and will therefore be summarised individually below. A UK-wide study by Parnham *et al*^60^ exploring access to targeted SFP reported that receiving an FSM was associated with increased odds of recently using a food bank^78^ but not reporting feeling hungry. In the month after the COVID-19 lockdown, 49% of eligible children did not receive any form of FSM. In this study, the sample size was small, and this may lead to chance findings or in ability to detect differences. A policy recommendation report by Guio^20^ explored evidence of the short-term and longer-term benefits of school meal provisions in EU countries. The report found that health outcomes are dependent on the programme take-up and quality of food. Additionally, while eating formally, pupils learn to be sociable and develop interaction skills. There are some findings on health outcomes associated with school meal provision, but these are more difficult to establish. School-based food and nutrition interventions were able to improve dietary behaviour, healthy eating and anthropometry, but the design of the intervention affects the magnitude of the effect. James^51^ used a cross-sectional design to investigate stigma associated with targeted provision and how this influences the peer effect. The study included 21 000 secondary school students in England. According to James,^51^ the presence of stigma dampens the peer effect and so students may not be benefiting from provision as much as possible. However, information about the provision has the opposite impact on the peer effect. These findings suggest that information is a more important part of the peer effect for those living in areas of greater deprivation and stigma is more important for those in the least deprived regions.

Long *et al*^52^ assessed the impacts of a universal system on meal cost and food quality in 508 US schools. This study finds that participation in the provision was associated with lower per-meal full cost with no differences in dietary quality. This indicates that in the USA, universal SFP can provide nutritious meals to more students without a financial disadvantage. Meier *et al*^47^ conducted a quantitative exploratory study including 576 parents. Completed in the USA, the study aimed to gain parents’ overall perceptions of the targeted school meals programme. This study found that parents of enrolled children were more likely to report positive perceptions of the school meals’ programme, whereas parents of children not receiving the programme were less likely to perceive the school meals programme in a positive light. Two limitations were noted that may have influenced results. First, parents of children not receiving provision made up much of the sample, and second, parents volunteered to participate in this survey; therefore, participation bias could have resulted from those who are more involved or have strong opinions about school lunch. A longitudinal study, using a difference-in-difference design by Lundborg *et al*^19^ evaluated the long-term impacts of the universal programme on children’s economic, educational and health outcomes throughout the life course. The study involved 1 529 760 participants from birth to age 50+. The study noted several findings; for instance, children exposed to the programme during their entire primary school period have 3% higher lifetime income. This effect was greater for pupils who were exposed at earlier ages and for pupils from poor households, suggesting that the programme reduced socioeconomic inequalities in adulthood. Additionally, a year of exposure to UFSM increased height growth and 9 years of school lunch exposure increased the likelihood of being of near perfect health. Exposure to school lunches also decreases the probability of being diagnosed with any health condition. The final study by Goodchild *et al*^59^ investigated factors associated with a universal infant FSM take-up and refusal in a multicultural urban community involving 676 parents of nursery-aged children (4–7 years). The study explored two groups: non-provision, n=159; or took provision, n=517. The non-provision group was more likely to be white British, have higher socioeconomic status, have English as a first language and involve their child in the decision over whether to take the school meal, compared with the provision group. It should be noted that the area the study was conducted is multicultural and school meals must cater for children from a variety of cultural backgrounds. Parents who did not complete questionnaires correctly were more likely to have lower socioeconomic status, be non-white British and have English as an additional language, meaning some groups were under-represented.

## Discussion

A scoping review methodology in accordance with the PRISMA guidelines^29^ was used to explore the global impacts of SFP on children’s health and well-being outcomes. To our knowledge, this is the first scoping review to comprehensively appraise SFP depending on SFP type (targeted or universal). To address our first research question, we found that most studies focused on physical health outcomes, particularly BMI and dietary intake. Emotional, psychological and social health were less commonly explored, and often through qualitative methods. Research was also concentrated in high-income countries, with limited representation globally. From a health perspective, SFP positively influenced children’s physical health, as well as their social, emotional and psychological well-being. Research on the effectiveness of SFP on BMI, weight and nutritional intake outcomes is limited but does indicate some positive impacts. Many rely on self-reported data, use small or context-specific samples, or apply inconsistent measures of emotional and psychological health. These limitations make it difficult to draw firm conclusions or compare findings across studies. As such, more robust and longitudinal research is needed to evaluate the impact of SFPs on children’s psychological and emotional outcomes. Both targeted and universal approaches play a crucial role in addressing inequalities among disadvantaged children by providing access to food; however, evidence supporting one as more effective in impacting health outcomes is limited. In terms of physical health, most included papers investigated BMI, with some conflicting findings. For example, most papers indicated positive impacts; however, two papers^6 58^ observed increased BMI under SFP. This discrepancy may be due to pre-existing malnutrition being present prior to provision or the foods served. For example, Goel *et al*^69^ found an increased sugar intake in free school breakfast. Though the nutritional quality of foods served differs between countries, making comparisons difficult, these differences may reflect broader inter-country inequities in funding, infrastructure and policy prioritisation of SFPs.^79^ SFPs appear to be a positive step forward in promoting positive psychological and emotional outcomes. However, studies in this area are limited and face several methodological challenges. Many rely on self-reported data, use small or context-specific samples, or apply inconsistent measures of emotional and psychological health. These limitations make it difficult to draw firm conclusions or compare findings across studies. As such, more robust and longitudinal research is needed to evaluate the impact of SFPs on children’s psychological and emotional outcomes. It is clear that more research is needed to evaluate the effectiveness of SFP on children’s psychological and emotional outcomes. SFP, whether universal or targeted, seems to ease food insecurity. Individuals from low socioeconomic backgrounds benefited more from SFP, regardless of whether they were targeted or universal. However, targeted programmes were associated with stigma, resulting in lower take-up and poorer mental health outcomes, emphasising the importance of implementing policies to mitigate stigma. Universal systems eliminated stigma and significantly increased uptake. Nonetheless, issues such as loss of agency and food quality persisted in influencing uptake rates. These findings reflect the key challenges and uptake factors identified across the literature in response to our second research question. They also highlight how the type of provision—universal or targeted—can shape children’s physical, psychological and social health outcomes, directly addressing our third research question.

### Implications for research

As previously mentioned, heterogeneity in research designs makes evaluating SFP difficult. This review also has limitations that should be addressed in future research. First, only English-language studies were included, which may have excluded relevant research published in other languages. Second, studies focusing solely on academic attainment were excluded, which may have limited insights into broader educational impacts of SFPs. While our review also aimed to capture a wide range of health impacts, studies focusing on anaemia were not identified through our search strategy. Most papers included in this review were conducted over a short time span. To fully understand the impacts of SFP on children’s health outcomes, longer term research designs are essential. Additionally, sociality was deemed an important aspect of FSM’s uptake, whereas stigma was associated with lower uptake. Therefore, future research should consider the implications of stigma on children’s health and how this can be alleviated. Only three studies investigated SFP as a means of addressing nutritional deficiencies among children. Future research should seek to explore these in more detail as well as other health concerns, such as respiratory illnesses and other common childhood illnesses associated with poor dietary intake.

### Implications for practice

There is not a definitive global recommendation for SFP implementation. Based on available evidence, both targeted and universal provision appear to be effective at improving children’s health outcomes to some degree; however, there are considerable limitations in current research. There is a notable need to address factors influencing uptake, particularly food quality and choice. Information was deemed important to parents when registering for SFP. Therefore, educational settings should ensure parents have sufficient information relating to SFP. Going forward, best practice and nutritional quality should be considered. Differences in outcomes across countries likely reflect variation in how SFPs are funded, implemented and integrated into broader education and welfare systems. For example, countries with universal provision models, such as Sweden and Finland, tend to report greater uptake and fewer stigma-related barriers,^80^ whereas targeted systems in countries like the UK and Poland may face challenges with reach and social equity.^63^

## Conclusions

Our review of 44 articles saw a diverse range of reported impacts of SFPs on children’s health outcomes, with variation across study contexts and programme types. SFP can contribute to healthy weight outcomes, although the nutritional benefits depend on the composition of the food offered. By synthesising evidence across physical, psychological, emotional and social health outcomes, rather than focusing on educational outcomes, it highlights the limited attention to children’s psychological and emotional well-being, the role of stigma in targeted programmes, and the advantages of universal provision for promoting equity. It also identifies a critical need for more evidence from low- and middle-income countries. Overall, both targeted and universal approaches contributed to decreasing food insecurity among children; however, universal approaches were more likely to reduce stigma and support greater uptake, offering additional benefits for equity.

## Supplementary material

10.1136/bmjopen-2024-093244 online supplemental file 1

10.1136/bmjopen-2024-093244 online supplemental file 2

10.1136/bmjopen-2024-093244 online supplemental file 3

## Data Availability

All data relevant to the study are included in the article or uploaded as supplementary information (Table S3). Extracted data are available upon reasonable request from the corresponding author.

## References

[1] World Food Programme (2022). State of school feeding worldwide 2022.

[2] Watkins K (2023). School meal programmes: a missing link in food systems reform prepared by the sustainable financing initiative (SFI) for school health and nutrition [internet]. https://www.edc.org/sites/default/files/School-meals-Food-Systems.pdf.

[3] Jansen PW, Roza SJ, Jaddoe VW (2012). Children’s eating behavior, feeding practices of parents and weight problems in early childhood: results from the population-based generation R Study. Int J Behav Nutr Phys Act.

[4] Hunter D, Giyose B, PoloGalante A Schools as a system to improve nutrition: a new statement for school-based food and nutrition interventions.

[5] World Health Organization (2006). Food and Nutrition Policy for Schools: A Tool for the Development of School Nutrition Programmes in the European Region.

[6] Vik FN, Van Lippevelde W, Øverby NC (2019). Free school meals as an approach to reduce health inequalities among 10–12- year-old Norwegian children. BMC Public Health.

[7] Cohen JFW, Hecht AA, McLoughlin GM (2021). Universal school meals and associations with student participation, attendance, academic performance, diet quality, food security, and body mass index: a systematic review. Nutrients.

[8] Destaw Z, Wencheko E, Kidane S (2022). Impact of school meals on educational outcomes in Addis Ababa, Ethiopia. Public Health Nutr.

[9] Chaudhary A, Sudzina F, Mikkelsen BE (2020). Promoting healthy eating among young people-a review of the evidence of the impact of school-based interventions. Nutrients.

[10] Khodaee GH, Emami Moghadam Z, Khademi G (2015). Healthy diet in children: facts and keys. Int J Pediatr.

[11] Ayton A, Ibrahim A (2019). The dramatic rise of ultra-processed foods. BMJ.

[12] Okunogbe A, Nugent R, Spencer G (2022). Economic impacts of overweight and obesity: current and future estimates for 161 countries. BMJ Glob Health.

[13] World Health Organization (2024). Obesity and overweight. https://www.who.int/news-room/fact-sheets/detail/obesity-and-overweight.

[14] World Health Organization WHO acceleration plan to stop obesity. https://iris.who.int/bitstream/handle/10665/370281/9789240075634-eng.pdf?sequence=1.

[15] Yue T, Zhang Q, Li G (2022). Global burden of nutritional deficiencies among children under 5 years of age from 2010 to 2019. Nutrients.

[16] Bringas Vega ML, Guo Y, Tang Q (2019). An age-adjusted EEG source classifier accurately detects school-aged barbadian childrenthat had protein energy malnutrition in the first year of life. Front Neurosci.

[17] Liu J, Qi X, Wang X (2022). Evolving patterns of nutritional deficiencies burden in low- and middle-income countries: findings from the 2019 global burden of disease study. Nutrients.

[18] Pasricha S-R, Tye-Din J, Muckenthaler MU (2021). Iron deficiency. The Lancet.

[19] Lundborg P, Rooth DO, Alex-Petersen J (2022). Long-term effects of childhood nutrition: evidence from a school lunch reform. Rev Econ Stud.

[20] Guio AC (2015). Free school meals for all poor children in Europe: an important and affordable target. Eur J Public Health.

[21] Guio AC, Frazer H, Marlier E Study on the economic implementing framework of a possible eu child guarantee scheme including its financial foundation: secondphase of the feasibility study for a child guarantee (FSCG2).

[22] Persson Osowski C, Fjellström C (2019). Understanding the ideology of the Swedish tax-paid school meal. Health Educ J.

[23] Hobbs G, Vignoles A (2010). Is children’s free school meal ‘eligibility’ a good proxy for family income?. British Educational Res J.

[24] Global Child Nutrition Foundation (2021). Poland: global survey of school meal programs 2021. https://gcnf.org/wp-content/uploads/2022/09/Poland_2021_final1.6.pdf.

[25] Global Child Nutrition Foundation (2021). Slovenia: Global Survey of School Meal Programs 2021.

[26] Lucas PJ, Patterson E, Sacks G (2017). Preschool and school meal policies: an overview of what we know about regulation, implementation, and impact on diet in the UK, Sweden, and Australia. Nutrients.

[27] Morelli CJ, Seaman PT (2005). Universal versus targeted benefits: the distributional effects of free school meals. Environ Plann C Gov Policy.

[28] Taylor A (2022). England’s free school meals scheme should be expanded to help more children living in poverty. BMJ.

[29] Moher D, Liberati A, Tetzlaff J (2009). Preferred reporting items for systematic reviews and meta-analyses: the PRISMA statement. PLoS Med.

[30] Locke A, James M, Jones H (2024). Impact of global school feeding programmes on children’s health and wellbeing outcomes: a scoping review protocol.

[31] Peters MDJ, Munn Z, Tricco AC (2020). Updated methodological guidance for the conduct of JBI scoping reviews incorporating the PCC framework. JBI Evidence Synthesis.

[32] Covidence systematic review software, veritas health innovation, Melbourne, Australia. www.covidence.org.

[33] Hong QN, Fàbregues S, Bartlett G (2018). The Mixed Methods Appraisal Tool (MMAT) version 2018 for information professionals and researchers. *EFI*.

[34] Hong QN, Gonzalez‐Reyes A, Pluye P (2018). Improving the usefulness of a tool for appraising the quality of qualitative, quantitative and mixed methods studies, the Mixed Methods Appraisal Tool (MMAT). J Eval Clin Pract.

[35] McArthur A, Klugárová J, Yan H (2015). Innovations in the systematic review of text and opinion. Int J Evid Based Healthc.

[36] Munn Z, Tufanaru C, Aromataris E (2014). JBI’s systematic reviews: data extraction and synthesis. AJN.

[37] Popay J, Roberts H, Sowden A (2006). Guidance on the conduct of narrative synthesis in systematic reviews. A product from the esrc methods programme version.

[38] Lunn J, Shaw D, De Bree A (2009). Leadership training for European nutritionists. Nutr Bull.

[39] Oostindjer M, Aschemann-Witzel J, Wang Q (2017). Are school meals a viable and sustainable tool to improve the healthiness and sustainability of children's diet and food consumption? A cross-national comparative perspective. Crit Rev Food Sci Nutr.

[40] Kitchen S, Tanner E, Brown V (2013). Evaluation of the Free School Meals Pilot.

[41] Bethmann D, Cho JI (2022). The impacts of free school lunch policies on adolescent BMI and mental health: evidence from a natural experiment in South Korea. SSM Popul Health.

[42] Holford A, Rabe B (2020). Impact of the Universal Infant Free School Meal Policy.

[43] Holford A, Rabe B (2022). Going universal: the impact of free school lunches on child body weight outcomes. J Public Econ Plus.

[44] Parnham JC, Chang K, Millett C (2022). The impact of the universal infant free school meal policy on dietary quality in english and scottish primary school children: evaluation of a natural experiment. Nutrients.

[45] Vik FN, Heslien KE, Van Lippevelde W (2020). Effect of a free healthy school meal on fruit, vegetables and unhealthy snacks intake in Norwegian 10- to 12-year-old children. BMC Public Health.

[46] Neervoort F, von Rosenstiel I, Bongers K (2013). Effect of a school feeding programme on nutritional status and anaemia in an urban slum: a preliminary evaluation in Kenya. J Trop Pediatr.

[47] Meier CL, Brady P, Askelson N (2022). What do parents think about school meals? An exploratory study of rural middle school parents’ perceptions. J Sch Nurs.

[48] Prado MAMB do, Francisco PMSB, Barros MB de A (2017). Uso de medicamentos psicotrópicos em adultos e idosos residentes em Campinas, São Paulo: um estudo transversal de base populacional*. Epidemiologia e Serviços de Saúde.

[49] Eustachio Colombo P, Patterson E, Elinder LS (2020). The importance of school lunches to the overall dietary intake of children in Sweden: a nationally representative study. Public Health Nutr.

[50] Horta PM, Carmo AS do, Junior EV (2019). Consuming school meals improves Brazilian children’s diets according to their social vulnerability risk. Public Health Nutr.

[51] James J (2012). Peer effects in free school meals: information or stigma? MWP 2012/11, max weber programme, European University Institute.

[52] Long MW, Marple K, Andreyeva T (2021). Universal free meals associated with lower meal costs while maintaining nutritional quality. Nutrients.

[53] Spence S, Matthews JN, McSweeney L (2021). Implementation of Universal Infant Free School Meals: a pilot study in NE England exploring the impact on Key Stage 1 pupil’s dietary intake. Public Health Nutr.

[54] Zailani H, Owolabi OA, Sallau AB (2023). Contribution of school meals to the recommended nutrient and energy intake of children enrolled in the National Homegrown School Feeding Program in Zaria, Nigeria. Arch Pediatr.

[55] Evans CE, Mandl V, Christian MS (2016). Impact of school lunch type on nutritional quality of English children’s diets. Public Health Nutr.

[56] Yang TC, Power M, Moss RH (2022). Are free school meals failing families? Exploring the relationship between child food insecurity, child mental health and free school meal status during COVID-19: national cross-sectional surveys. BMJ Open.

[57] Davis W, Kreisman D, Musaddiq T (2023). The effect of universal free school meals on child BMI. SSRN Journal.

[58] Altindag DT, Baek D, Lee H (2020). Free lunch for all? The impact of universal school lunch on student misbehavior. Econ Educ Rev.

[59] Goodchild GA, Faulks J, Swift JA (2017). Factors associated with universal infant free school meal take up and refusal in a multicultural urban community. J Hum Nutr Diet.

[60] Parnham JC, Laverty AA, Majeed A (2020). Half of children entitled to free school meals did not have access to the scheme during COVID-19 lockdown in the UK. Public Health.

[61] Zuercher MD, Orta-Aleman D, Cohen JFW (2024). The benefits and challenges of providing school meals during the first year of California’s Universal school meal policy as reported by school foodservice professionals. Nutrients.

[62] Jessiman PE, Carlisle VR, Breheny K (2023). A qualitative process evaluation of universal free school meal provision in two London secondary schools. BMC Public Health.

[63] Carlisle VR, Jessiman PE, Breheny K (2023). A mixed methods, quasi-experimental evaluation exploring the impact of a secondary school universal free school meals intervention pilot. IJERPH.

[64] Hecht AA (2022). Universal Free School Meals: Implementation of the Community Eligibility Provision and Impacts on Student Nutrition, Behavior and Academic Performance [Dissertation].

[65] Taylor J, Garnett B, Horton MA (2020). Universal free school meal programs in vermont show multi-domain benefits. J Hunger Environ Nutr.

[66] Yamaguchi M, Kondo N, Hashimoto H (2018). Universal school lunch programme closes a socioeconomic gap in fruit and vegetable intakes among school children in Japan. Eur J Public Health.

[67] Cardoso SG, Truninger M, Ramos V (2019). School meals and food poverty: children’s views, parents’ perspectives and the role of school. Child Soc.

[68] Goel NJ, Caccavale LJ, Mazzeo SE (2019). Total sugar in free breakfasts served in Virginia elementary schools. Health Behav Policy Rev.

[69] Illøkken KE, Johannessen B, Barker ME (2021). Free school meals as an opportunity to target social equality, healthy eating, and school functioning: experiences from students and teachers in Norway. Food Nutr Res.

[70] McKelvie-Sebileau P, Swinburn B, Glassey R (2023). Health, wellbeing and nutritional impacts after 2 years of free school meals in New Zealand. Health Promot Int.

[71] Sahota P, Woodward J, Molinari R (2014). Factors influencing take-up of free school meals in primary- and secondary-school children in England. Public Health Nutr.

[72] Chelius C, Bacon KA, Orta-Aleman D (2025). California middle and high school students report wanting fresh and healthy school lunch in the context of Universal School Meals. J Nutr Educ Behav.

[73] Mauer S, Torheim LE, Terragni L (2022). Children’s participation in free school meals: a qualitative study among pupils, parents, and teachers. Nutrients.

[74] Rahim N, Kotecha M, Callanan M (2012). Implementing the Free School Meals Pilot.

[75] Garton K, Riddell C, McKelvie-Sebileau P (2023). Not Just a Free Lunch a logic model and evidence review for the Ka Ora, Ka Ako | Healthy School Lunch programme. Pq.

[76] Chambers S, Dundas R, Torsney B Civic engagement.

[77] Chambers S, Boydell N, Ford A (2020). Learning from the implementation of Universal Free School Meals in Scotland using Normalisation Process Theory: lessons for policymakers to engage multiple stakeholders. Food Policy.

[78] The Trussell Trust (2024). Emergency food- the trussell trust. https://www.trusselltrust.org/get-help/emergency-food/.

[79] Béné C, Fanzo J, Prager SD (2023). Food systems policy research: a systematic scoping review of policy effectiveness, trade-offs, synergies and barriers for nutrition, health and sustainability. Food Secur.

[80] Gallegos D, Manson A, Vidgen H (2025). School-provided meals and the prevention of childhood obesity. Curr Obes Rep.

