# Supplementary material for: Impacts of Global School Feeding Programmes on Children’s Health and Wellbeing Outcomes: A Scoping Review
Source: BMJ Open. 2025 Oct 2;15(10):e093244. doi: 10.1136/bmjopen-2024-093244 (PMC12496081; doi:10.1136/bmjopen-2024-093244)
Supplement: online supplemental file 1 [file bmjopen-15-10-s001.docx]

# Supplementary File: Full Search Strategy

This document provides the full search strategy used in the scoping review titled 'Impacts of Global School Feeding Programmes on Children’s Health and Wellbeing Outcomes'. The strategy follows PRISMA-ScR guidelines to ensure transparency and reproducibility. Full protocol can be found at: Locke A, James M, Jones H, Davies R, Williams F, Brophy S. Impact of Global School Feeding Programmes on Children’s Health and Wellbeing Outcomes: A Scoping Review Protocol 2024. doi:10.17605/OSF.IO/UZRWD.

## Databases Searched:

- PubMed
- Medline
- Web of Science
- Google Scholar (including Science Direct, ProQuest Central, EBSCO, CINAHL via linked results)

## Search Dates:

- Initial search conducted: December 2023
- Updated search conducted: 7 July 2025

## Search Strings:

("Universal Free School Meals" OR "School Feeding Programmes" OR "Free School Meals") AND ("Children" OR "School Children" OR "Adolescents") AND ("Health" OR "Wellbeing" OR "Nutrition" OR "Dietary Intake") AND ("Targeted" OR "Universal" OR "Policy" OR "Provision") AND ("Impacts of" OR "Effects of" OR "Outcomes of")

Alternative phrases used in broader searches included:

- "School Meal Provision"
- "Challenges with School Feeding Programmes"
- "School Meals and Emotional Health"
- "School Feeding AND Social Health"

## Filters Applied:

- Language: English only
- Publication Date: 1 January 2009 to 7 July 2025
- Population: Children aged 5–16
- Study Types: Peer-reviewed empirical studies (quantitative, qualitative, or mixed-methods), case studies, grey literature (reports, opinion pieces), and reviews.

## Search Notes:

- Google Scholar results were screened up to the first 100 entries per search string.
- Reference lists of included articles were also screened for additional studies.
- Study design filters were not applied during the database search to capture the full range of eligible designs in line with the scoping review methodology.

## Search Management Tools:

- Mendeley for citation management.
- Covidence for screening and deduplication.
