# Supplementary material for: Impacts of Global School Feeding Programmes on Children’s Health and Wellbeing Outcomes: A Scoping Review
Source: BMJ Open. 2025 Oct 2;15(10):e093244. doi: 10.1136/bmjopen-2024-093244 (PMC12496081; doi:10.1136/bmjopen-2024-093244)
Supplement: online supplemental file 2 [file bmjopen-15-10-s002.docx]

| **Title** | **Author** | **Year** | **Country** | **Provision** | **Sample Size** | **School age** | **Outcome Measured** | **Design** | **Results** | **Limitations/Gaps** | **Recommendations** |
| --- | --- | --- | --- | --- | --- | --- | --- | --- | --- | --- | --- |
|  |  |  |  |  |  |  |  |  |  |  |  |
