# Supplementary material for: Impacts of Global School Feeding Programmes on Children’s Health and Wellbeing Outcomes: A Scoping Review
Source: BMJ Open. 2025 Oct 2;15(10):e093244. doi: 10.1136/bmjopen-2024-093244 (PMC12496081; doi:10.1136/bmjopen-2024-093244)
Supplement: online supplemental file 3 [file bmjopen-15-10-s003.docx]

| **Study** | **Country** | **Design** | **Sample** | **Quality** | **Provision Type** | **School Year** | **Outcome Measures** | **Summary** |
| --- | --- | --- | --- | --- | --- | --- | --- | --- |
| Persson & Fjellström [22] | Sweden | Article Piece | Not Specified | High | Universal | All | Health, dietary intake, habits. | Positive: promoting healthy habits and improving dietary intake. |
| Guio [20] | Europe | Policy Recommendation | N/A | High | Other | N/A | Benefits of SFP across Europe | Positive: School meals can support healthy eating habits and improve dietary behaviour. Improved social skills. |
| Harper and Wood [38] | UK Wide | Report | N/A | High | Targeted | Primary | Take-up, Stigma, changes in eating habits (at home and school). Parent and student views of FSM. | Positive: Improved eating habits for those in low-income households, increased uptake and reduced stigma. |
| Oostindjer et al. [39] | Cross-National | Discussion/Essay | N/A | High | Other | N/A | The role of school meals in shaping food behaviours and improving population health sustainably across countries | Negative: Removing meat and fish from school meals reduced participation and increased food waste. Lack of clear goals limits the broader societal impact of school meal programs. |
| Kitchen et al. [40] | England | Impact Report | Not specified | Moderate | Universal | All | Impact on; Diet, health, and behaviour.  Impact on take-up. | Mixed: UFSM increased take-up and shifted pupils' lunchtime choices toward healthier hot meals, especially in primary schools, but no significant health benefits (e.g., BMI changes) were observed |
| Lundborg et al. [19] | Sweden | Difference-in-Difference | 1,529,760 | High | Universal | Birth to 50+ | Long term impact of the SFP program on children’s economic, educational, and health outcomes throughout life. | Positive: Long-term UFSM exposure improves health outcomes and lifetime income, particularly for low-income pupils. |
| Bethmann and Cho [41] | South Korea | Difference-in-Difference | Not specified | High | Universal | Aged 9-16 | Student BMI and mental health status. | Positive: Free school lunches improve BMI, mental health, and overall student welfare. |
| Holford and Rabe [42] | England | Difference-in-Difference | Not specified | Low | Universal | Primary | Impact on household expenditure, uptake and weight outcomes. | Positive: SFP increased meal uptake, reduced household food costs, and improved healthy weight status. |
| Holford and Rabe [43] | England | Difference-in-Difference | 153,522 | High | Universal | Primary | Impact of switching to universal free school meals on young children’s bodyweight. | Positive: Longer exposure to UIFSM improves bodyweight outcomes, increases the likelihood of children being a healthy weight, and significantly boosts meal take-up among those not previously eligible. |
| Parnham et al. [44] | England & Scotland | Difference-in-Difference | 854 | High | Universal | Primary | Impact of the SFP policy on dietary intakes. | Positive: Increased uptake and reduced intake of less healthy packed lunch foods. |
| Vik et al. [6] | Norway | Nonrandomised Control Trial | 164 | High | Targeted | Secondary | Dietary habits at lunchtime (food frequency), BMI and waist circumference. As well as moderating effects of socio-economic status. | Mixed: Increased healthy food intake, especially among lower socio-economic groups, but also led to a rise in BMI with no change in waist circumference. |
| Vik et al. [45] | Norway | Nonrandomised Control Trial | 164 | High | Universal | Secondary | To investigate whether serving a free healthy school meal for one year resulted in a higher intake of fruit and vegetables and a lower intake of unhealthy snacks. | Positive: Free healthy school meals increased vegetable intake, though they did not reduce unhealthy snack consumption. |
| Neervoort et al. [46] | Kenya | Nonrandomised Control Trial | 67 | Low | Universal | Primary | Anaemia, stunting, wasting and malnutrition. | Positive: The programme reduced anaemia and malnutrition, and improved child growth in the study group. |
| Meier et al. [47] | USA | Quantitative Exploratory | 576 | Low | Targeted | N/A | Parental perceptions of SFP. | Positive: Parents of participating children reported more positive perceptions of the school meals program. |
| Batista et al. [48] | Brazil | Cross-Sectional | 7,017 | Moderate | Targeted | Aged 4-14 | Prevention of overweight through school meals programme. | Negative: High rates of overweight and high intake of ultra-processed foods were observed among schoolchildren. |
| Colombo et al. [49] | Sweden | Cross-sectional | 2002 | High | Universal | Secondary | Dietary intake. | Positive: School meals significantly contribute to weekday nutrient intake and are more nutritious than meals eaten outside of school. |
| Horta et al. [50] | Brazil | Cross-sectional – Analytical | 1357 | High | Targeted | Primary | Impacts of SFP on Vulnerability risk and dietary intake. | Positive: School meals improve children's diets and help reduce dietary inequalities, especially in socially vulnerable groups. |
| James [51] | England | Cross-sectional - Descriptive | 21,000 | Low | Targeted | Secondary | Stigma associated with SFP Provision. | Mixed: Information boosts positive peer effects on school meal uptake, especially in deprived areas, while stigma reduces uptake, particularly in less deprived regions. |
| Long et al. [52] | USA | Cross-sectional | 508 (schools) | High | Universal | All | Meal cost and food quality. | Positive: UFMs provide nutritious meals at lower cost per meal, with no negative impact on dietary quality. |
| Spence et al. [53] | England | Cross-sectional | 196 | Moderate | Universal (Infant) | Primary | pre and post universal SFP on dietary intake. | Mixed: UIFM reduced intake of added sugars but increased consumption of cakes and puddings at lunch. |
| Zailani et al. [54] | Nigeria | Cross-sectional | 315 | High | Targeted | Primary | Portion sizes of school meals, nutrient intake. | Mixed: SFP meals met key nutrient requirements but lacked important food groups like fruits, meat, poultry, and fish. |
| Evans [55] | England | Cross-sectional -Observational | 2709 | High | Other | Primary | Nutritional and dietary differences by lunch type (School meal Vs Packed lunch) | Positive: School meals support healthier diets with lower sugar intake, more vegetable variety, and higher water consumption compared to packed lunches. |
| Yang et al. [56] | UK | Cross-sectional | 2166 | High | Targeted | All | Food security status and school meal status. | Negative: Food insecurity is linked to poor mental health, and receiving FSM did not mitigate this risk among food-insecure children. |
| Davis et al. [57] | Georgia- USA | Descriptive | Not specified | Low | Universal | All | BMI - weight and height. | Mixed: School meal eligibility did not raise obesity risk, but students in community  Eligibility Program (CEP) schools showed higher average BMI compared to non-CEP schools. |
| Altindag et al. [58] | South Korea | Empirical analysis | 8000 (schools) | Moderate | Universal | All | means-tested SFP and Universal on student behaviour - misbehaviour, bullying, and violence in schools. | Positive: Universal school meals reduced stigma and led to a 35% drop in behavioural incidents, particularly physical fights, despite no change in nutritional intake. |
| Goodchild et al. [59] | England | Cross-sectional - Descriptive | 676 | Moderate | Universal | Primary | Impact of universal SFP on health and diet. | Neutral: No direct impact on health or diet was reported. |
| Parnham et al. [60] | UK | Cross-sectional | 635 | Low | Targeted | All | SFP eligibility and food insecurity. | Negative: FSM access was inconsistent during COVID-19, with nearly half of eligible children missing out, and increased food bank use among recipients, especially in Wales. |
| Zuercher et al. [61] | USA | Cross-sectional | 430 | High | Universal | All | Meal participation, stigma and meal quality. | Positive: UFSM increased student participation and reduced stigma around school meal provision. |
| Jessiman et al [62] | London | Mixed Methods | 36 | High | Universal | Secondary | Access to a healthy meal, food insecurity and nutritional intake. | Positive: SFP in secondary schools was widely seen as beneficial, promoting equality, reducing financial burden, and improving access to meals. |
| Hecht [63] | USA | Mixed methods | 69,832 | High | Universal | All | Student meal participation, behaviour, and academic outcomes. | Positive: Reduced hunger and stigma, and may improve physical and mental health, behaviour, academic outcomes, and school climate. |
| Taylor et al. [64] | Vermont,USA | Mixed methods | 116 | Moderate | Universal | Staff | School climate, behaviour and attainment | Positive: Improved school social climate, reduced stigma and stress, eased financial strain, and fostered a more inclusive school community, with strong support from staff for its implementation. |
| Yamaguchi et al. [65] | Japan | Mixed methods | 719 | High | Universal | Primary | Fruit and veg intake, socio-economic status. | Positive: Universal school lunches support fruit and vegetable intake and help reduce dietary gaps across socio-economic groups. |
| Cardoso et al. [67] | Portugal | Qualitative | 88 | High | Targeted | Secondary | Children and parents’ perspectives of food at school. | Mixed: School meals support dietary variety and ease family budgets, but concerns about meal quality affect uptake, especially among children not receiving free or discounted meals. |
| Goel et al. [68] | USA - Virginia | Observational | 35(Schools) | Moderate | Other | Primary | Sugar intake in FS breakfast. | Negative: School meals may contribute to excessive sugar intake among children. |
| Illøkken et al. [69] | Norway | Qualitative | 18 | Low | Targeted | Secondary | Students’ and teachers’ experiences of receiving free school meals. | Positive: Free school meals promote healthy behaviours, social interaction, skill development, school functioning, and greater social equality. |
| McKelvie-Sebileau1et al. [70] | New Zealand | Qualitative | 56 | High | Universal | Secondary | Health, Wellbeing and nutritional impacts. Uptake. | Mixed: Improved food security, wellbeing, and nutritional knowledge, but challenges included low uptake, food waste, poor communication, and student preference for less healthy options. |
| Sahota et al. [71] | England | Qualitative | 116 | Moderate | Targeted | All | Factors influencing up-take. | Positive: FSM uptake was influenced by food quality and social factors, with stigma reduced through cashless systems and improved lunchtime environments supporting social interaction. |
| Chelius et al. [72] | USA | Qualitative | 67 | High | Universal | All | Perceptions of school lunch (taste, freshness, healthfulness, preferences). Up-take of provision. | Positive: Increased participation, reduced stigma, and normalized school meals, though meal quality remained key to student uptake. |
| Mauer et al. [73] | Norway | Qualitative | 66 | High | Targeted | Secondary | Factors influencing students’ participation. | Mixed: Social time important to students. unique food options encouraged school meal participation, but competition from local food outlets limited uptake. |
| Carlisle et al. [74] | London | Qualitative | 1255 | High | Universal | Not specified | Hunger/nutrition, Family finances and food insecurity, educational impacts, stigma/shame, social and emotional impacts. | Mixed: UFSM was perceived to improve social skills, behaviour, and household food security, but concerns about food quality and unhealthy choices remained. Qualitative data did not indicate changes in health outcomes. |
| Rahim et al. [75] | England | Government Report | 65 (schools) | High | Universal & Targeted | Primary | Dietary preferences, Health, Social Skills, behaviour. | Positive: Universal provision improved take-up, dietary variety, social skills, and some health outcomes. |
| Garton et al. [76] | New Zealand | Rapid Narrative Review | Not specified | High | Universal | Not specified | International evidence on what universal SFP can achieve. | Positive: Universal provision reduced hunger, improved physical and mental wellbeing, lowered inequalities, and supported better health, education, and economic outcomes. |
| Chambers et al. [77] | Scotland | Qualitative Case Study | 36,068 | Low | Universal | N/A | Characteristics associated with uptake. | Positive: Uptake increases with reduced stigma, better food quality, and social dining with friends. |
| Chambers et al. [78] | Scotland | Case Study | 29 | High | Targeted | Secondary | Examining the implementation of SFP in Scotland. | Positive: SFP supported low-income working families, promoted healthier eating habits, and offered higher-quality, more varied meals than those from home. |
