# Supplementary material for: Impacts of Global School Feeding Programmes on Children’s Health and Wellbeing Outcomes: A Scoping Review
Source: BMJ Open. 2025 Oct 2;15(10):e093244. doi: 10.1136/bmjopen-2024-093244 (PMC12496081; doi:10.1136/bmjopen-2024-093244)
Supplement: Supplementary file 4 [file bmjopen-2024-093244.reviewer_comments.pdf]

PEER REVIEW HISTORY

BMJ Open publishes all reviews undertaken for accepted manuscripts. Reviewers are asked to complete a checklist review form and are provided with free text boxes to elaborate on their assessment. These free text comments are reproduced below.

ARTICLE DETAILS

Title (Provisional)

Impacts of Global School Feeding Programmes on Children's Health and Wellbeing Outcomes: A Scoping Review

Authors

Locke, Amy; James, M; Jones, Hope; Davies, Rachel; Williams, Francesca; Brophy, Sinead

---

VERSION 1 - REVIEW

**Reviewer**	**1**
**Name**	**Monteith, Hiliary**
**Affiliation**	**University of Toronto, Nutritional Sciences**
**Date**	**04-Nov-2024**
**COI**	**None**

Thank you for your thorough scoping review. You describe your protocol in detail and did well to reference each of the papers throughout your results section. I do have some concerns with the clarity of this work and think that some revisions would strengthen the impact of your work. Generally, some sentences require rewording to support clarity. There are a few times in which you mention the impact of SFP on child poverty, food insecurity, and malnutrition; however, these sentences require rewording as they don't consider some of the nuance that exists within the current literature on these topics. I also recommend providing more context for the specific countries included in this review and ensuring that your introduction and discussion reflect the global health context. I provide detailed comments in the attached file.

**** The reviewer provided a marked copy with additional comments. Please contact the publisher for full details. ****

**Reviewer**	**2**
**Name**	**Dokova, Klara**
**Affiliation**	**Medical University of Varna, Varna, Bulgaria**

**Date**	**01-Jul-2025**
**COI**	**None**

The work of the authors is substantive and on a very important public health topic.

Comments for the Abstract:

The introduction leads toward 2 questions:

1. "Lack of information on the effectiveness of school feeding programmes (SFPs) in terms of what are their effects on child health outcomes"

This implicitly indicates the need for the assessment of the effectiveness of SFPs, which is not compatible with the design of the study – scoping review is not suitable for assessing effectiveness.

2. "Whether school feeding programmes should be universal or targeted"

The Aim is defined as: to…" explore global research on school feeding programmes, examine what health outcomes they are interested in and examine what policies they follow: targeted or universal…" This is different from examining/assessing the effectiveness of the programmes.

So the research aim does not become very clear from the abstract.

Methods - Do not mention eligibility criteria- required.

Results: the period 2009-2024 as stated includes 16 years and not 15 as mentioned?

Results and Conclusion: Targeted school feeding programmes – less stigma this is not directly related to health? SFP – better child weight?

The abstract does not specify the aim clearly. So the chosen design is questionable.

The methods and results are insufficiently presented.

Main text:

Figure 1 After eligibility assessment – wrong outcomes, wrong intervention….

Why using the word wrong, if it is just not fulfilling the search criteria?

Some imprecisions related to the references:

No 4 – insufficient details given.

No 19 – not very relevant as evidence in the sentence where it is cited first. Leave only ref. 20.

No 21 – not very relevant as evidence in the sentence where it is cited first.

Rows 13-14: No reference for Slovenia and Poland? It will be good to add relevant ones.

Three research questions (that is a lot and they are too broad and thus later insufficiently answered):

1. To what extent does the existing literature examine the impacts of school feeding programmes on the physical, emotional, psychological, and social health of

school children globally and

2. What challenges, including factors influencing uptake, are reported in the literature regarding the implementation of free school meal provision and

3. Does the effectiveness of SFP on children's health outcomes vary depending on the SFP policy (targeted or universal).

The greatest problem with the manuscript is its extremely wide scope. The authors are interested in the effects of school feeding programmes on the physical emotional, psychological and social aspects of health which is already too mush. They are further interested in all sorts of study design which further complicates their task.

The topic is very wide – too many and too wide outcomes. So it is difficult to write specific inclusion criteria. The presented inclusion criteria are too wide, especially the first one: Children's physical health – Illness, physical activity, and/or diet dietary intake - each one is sufficient for a review.

So the used key words are hardly covering the wide inclusion criteria. E.g. physical / anthropometric status and / or its elements e.g. BMI are not covered with the key words?

And there are a number of missing relevant publications in that group e.g.: doi:10.1001/jamapediatrics.2022.5828; https://doi.org/10.1017/jns.2022.73

And many more.

The same with the publications on school feeding programmes and anaemia? None is included in the reviewed manuscript and that is an issue directly related to children's health?

This important aspect of the reviewed manuscript needs greater attention and precision.

The search terms and inclusion criteria have to be further specified. The search strategy is hardly reproducible.

Classification in terms of age seems to miss two studies (9+12+10+2+1+1+5=40?)

The first reported results are actually related to BMI and weight and obesity – which was not expected based on the description of the inclusion criteria and the key words.

The results are presented narratively without quantitative and statistical evidence and often clarity from what study design they are extracted thus making them not convincing and not interpretable.

It will be much better if the results can be summarised in table/s format, including information on design, sample size, quality and some way of presenting / summarising the results (positive/negative impact on the various health aspects).

The discussion is superficial.

It lacks depth in relation to the enormous number of publications on the topic.

The conclusion mentions 44 articles while the methods and the results section claim 42 articles were reviewed. The conclusions does not add important new knowledge to what is already known from the existing scientific literature.

VERSION 1 - AUTHOR RESPONSE

Reviewer: 1
Thank you for your thorough scoping review. You describe your protocol in detail and did well to reference each of the papers throughout your results section. I do have some concerns with the clarity of this work and think that some revisions would strengthen the impact of your work. Generally, some sentences require rewording to support clarity. There are a few times in which you mention the impact of SFP on child poverty, food insecurity, and malnutrition; however, these sentences require rewording as they don't consider some of the nuance that exists within the current literature on these topics. I also recommend providing more context for the specific countries included in this review and ensuring that your introduction and discussion reflect the global health context. I provide detailed comments in the attached file.

Reviewer: 2
Comments for the Abstract:
The introduction leads toward 2 questions:
1. "Lack of information on the effectiveness of school feeding programmes (SFPs) in terms of what are their effects on child health outcomes"
This implicitly indicates the need for the assessment of the effectiveness of SFPs, which is not compatible with the design of the study – scoping review is not suitable for assessing effectiveness.

Thank you for this observation. We have revised the language in the Abstract and Introduction to avoid implying an assessment of effectiveness and to better reflect the aims and scope of a scoping review.

2. "Whether school feeding programmes should be universal or targeted"

The Aim is defined as: to…" explore global research on school feeding programmes, examine what health outcomes they are interested in and examine what policies they

follow: targeted or universal…" This is different from examining/assessing the effectiveness of the programmes.
So the research aim does not become very clear from the abstract.

Thank you for highlighting this issue. We agree that the original abstract did not clearly distinguish between exploring the effectiveness of school feeding programmes and our actual aim, which is to map and characterise global research on these programmes. In response, we have revised the abstract to clarify that the review does not assess effectiveness. It now reads:

"This scoping review aims to map global literature on school feeding programmes, identify which health and wellbeing outcomes are reported, and explore how these outcomes vary by programme type (targeted vs universal)."
(Page 2, Lines 30-32).

Methods - Do not mention eligibility criteria- required.
Results: the period 2009-2024 as stated includes 16 years and not 15 as mentioned?

Thank you for your comment. To clarify, the manuscript specifies the correct search period as 2009 to 2023, covering 15 years. This has now been updated to reflect the latest search, covering 2024 to 2025. (Page 9, Lines 215-217).

Results and Conclusion: Targeted school feeding programmes – less stigma this is not directly related to health? SFP – better child weight?

While stigma is not a direct physical health outcome, it is closely linked to psychological and emotional wellbeing, which we included within our broader definition of health for this review. Similarly, child weight was considered under physical health.

The abstract does not specify the aim clearly. So the chosen design is questionable. The methods and results are insufficiently presented.

Thank you for your feedback. We have revised the abstract to more clearly reflect the aim of the review and to align it with the scoping review methodology. The wording has been updated to emphasise the mapping of existing literature rather than assessing effectiveness.

Main text:
Figure 1 After eligibility assessment – wrong outcomes, wrong intervention….

Why using the word wrong, if it is just not fulfilling the search criteria?

Thank you for this observation. We used the term “wrong outcomes” and “wrong intervention” in line with the standard PRISMA diagram conventions to summarise reasons for exclusion. For example, wrong outcomes were used where the papers did not measure the outcomes we intended to explore i.e., children’s health outcomes.

Some imprecisions related to the references:
No 4 – insufficient details given.

Thank you for this comment. We have revised the text in the Introduction to provide more context for reference [4], clarifying that it refers to a global statement on school-based food and nutrition systems. This has been reflected in the updated sentence referencing Hunter et al. [4]. (Page 3, Lines 76-79).

No 19 – not very relevant as evidence in the sentence where it is cited first. Leave only ref. 20. No 21 – not very relevant as evidence in the sentence where it is cited first.

References no.19 and 21 have now been removed from the manuscript.

Rows 13-14: No reference for Slovenia and Poland? It will be good to add relevant ones.

A reference for both Poland and Slovenia has been added - See ref’s no. 24 & 25. (Page 8, Line 128).

Three research questions (that is a lot and they are too broad and thus later insufficiently answered):
1. To what extent does the existing literature examine the impacts of school feeding programmes on the physical, emotional, psychological, and social health of school children globally and

We agree that Research Question 1 is broad, reflecting the scoping nature of the review. To improve clarity, we have now added a brief summary at the start of the Discussion section to explicitly address the extent to which different health outcomes and global contexts are represented in the existing literature. Specifically, we note that:

“To address our first research question, we found that most studies focused on physical health outcomes, particularly BMI and dietary intake. Emotional, psychological, and social health were less commonly explored, and often through qualitative methods. Research was also concentrated in high-income countries, with limited representation globally.”
(Page 30, Lines 667 - 670).

2. What challenges, including factors influencing uptake, are reported in the literature regarding the implementation of free school meal provision and

3. Does the effectiveness of SFP on children’s health outcomes vary depending on the SFP policy (targeted or universal).

Thank you. We have added sentences to the Discussion to explicitly address Research Questions 2 and 3, highlighting key challenges to uptake and how the type of provision can shape children’s health outcomes. For example:

“SFP, whether universal or targeted, seems to ease food insecurity. Individuals from low socio-economic backgrounds benefited more from SFP, regardless of whether they were targeted or universal. However, targeted programs were associated with stigma, resulting in lower take-up and poorer mental health outcomes.”
(Page 32, Lines 697 - 700).

The greatest problem with the manuscript is its extremely wide scope. The authors are interested in the effects of school feeding programmes on the physical emotional, psychological and social aspects of health which is already too mush. They are further interested in all sorts of study design which further complicates their task.

This review was designed to be intentionally broad and exploratory in nature, as the topic has not been comprehensively examined in previous literature. Our aim was to capture a wide range of outcomes and implementation approaches related to school feeding programmes globally. By adopting a broad scope, we sought to gain a holistic understanding of how these programmes are structured, delivered, and experienced across different contexts and regions. The primary focus of this review is on the implementation of school feeding initiatives rather than on limiting the analysis to a narrow set of predefined outcomes. While we recognise the value of more targeted investigations, such as those focusing on specific health, educational, or economic impacts, these narrower scopes may be more appropriate

for future research building upon the findings of this initial review.
A scoping review aims to map the breadth of evidence available on a topic and summarise existing literature, this is often as a first step towards a more focussed systematic review or to inform future research direction and scope.

The topic is very wide – too many and too wide outcomes. So it is difficult to write specific inclusion criteria. The presented inclusion criteria are too wide, especially the first one: Children's physical health – Illness, physical activity, and/or diet dietary intake - each one is sufficient for a review.

Thank you very much for this comment. We agree that the scope of our review is broad, reflecting its scoping nature. To address your concern, we have clarified in the manuscript that while our inclusion criteria were intentionally wide to capture the diverse ways school feeding programmes impact children's health, our primary focus was on outcomes related to children's weight and general illness associated with weight (rather than specific diseases or health conditions), physical activity, and overall diet intake. This clarification ensures that the breadth of the review remains appropriate for identifying patterns and gaps across the literature.

So the used key words are hardly covering the wide inclusion criteria. E.g. physical / anthropometric status and / or its elements e.g. BMI are not covered with the key words? And there are a number of missing relevant publications in that group e.g.: doi:10.1001/jamapediatrics.2022.5828; https://doi.org/10.1017/jns.2022.73 And many more.

We acknowledge that, despite using broad and inclusive search terms, there may be relevant publications that were not identified through our search strategy. This is a common limitation in reviews of this nature, particularly when aiming to capture a wide and diverse body of literature. However, we believe that the general exploratory approach taken in this review remains valuable, as it allows us to map key trends, identify common implementation strategies, and highlight areas of convergence and divergence across global school feeding programmes. It is important to note that the primary objective of this review was not to conduct an exhaustive analysis of all issues related to child health. Rather, our focus was on understanding how school feeding programmes are implemented in different contexts, with the intention of providing a foundation for future, more targeted investigations. While child health is undoubtedly an important consideration, a detailed examination of every health-related outcome was beyond the scope of this review.

The same with the publications on school feeding programmes and anaemia? None is included in the reviewed manuscript and that is an issue directly related to

children’s health? This important aspect of the reviewed manuscript needs greater attention and precision.

Thank you for your comment, please see response above relating to specific illnesses.

The search terms and inclusion criteria have to be further specified. The search strategy is hardly reproducible.

Thank you for this suggestion. We have clarified the focus of the research within the manuscript.

Classification in terms of age seems to miss two studies (9+12+10+2+1+1+5=40?)

This section has been re-done to include all 44 papers.

The first reported results are actually related to BMI and weight and obesity – which was not expected based on the description of the inclusion criteria and the key words.

Thank you for your comment. The purpose of this scoping review was to map the existing literature on school feeding programmes and their reported impacts on children’s health and wellbeing. As such, we did not include specific terms like “BMI” in the search strategy, since the aim was to capture a broad range of outcomes without assuming which health indicators had been studied. BMI and weight outcomes fall under our predefined category of physical health and were therefore eligible for inclusion. These outcomes were also among the most frequently reported in the included studies, which is why they were presented first in the Results section.

The results are presented narratively without quantitative and statistical evidence and often clarity from what study design they are extracted thus making them not convincing and not interpretable.

Thank you. We have now clarified the study designs and looked to bring similar studies together, but the level of heterogeneity means we could only undertake a narrative synthesis. This has been clarified in in the manuscript:

“Data analysis consisted of a narrative synthesis due to heterogeneity of both study designs and outcomes among included studies, making statistical meta-analysis impractical.”
(Page 10, Lines 249 - 250).

It will be much better if the results can be summarised in table/s format, including information on design, sample size, quality and some way of presenting / summarising the results (positive/negative impact on the various health aspects).

Thank you for the suggestion. We have created a summary table including study design, sample size, provision type, quality rating, measured outcomes and summarised findings. The table is referenced in the Results section.

Study (Author, Year)	Country	Design	Sample	Quality	FSM Type	Age	Outcome Measures	Summary
Vik et al. (2019)	Norway	Non-randomised Control	164	High	Universal	10-12	Behaviour/ Education/ Height/Weight/Waist Circumference	Positive: UFSM may contribute to promoting healthy eating and reduce inequalities

The discussion is superficial. It lacks depth in relation to the enormous number of publications on the topic.

Thank you for your feedback. We would like to clarify that this review is the first of its kind to explore the global implementation of school feeding programmes in a broad and systematic manner. It was intentionally designed as an exploratory study to map out how different countries are approaching school feeding strategies, particularly in light of the growing interest and policy shifts occurring in the UK. As the UK moves towards the expansion of universal free school meals, there is a timely need to understand how such programmes are being delivered internationally. This includes the models adopted, challenges faced, and outcomes observed. This global perspective is intended to inform future evaluations and decision-making within the UK context. While the review does not provide an in-depth analysis of specific outcomes, it lays important groundwork for more focused research in the future. As no other reviewers have raised this concern, we believe the current approach remains appropriate.

The conclusion mentions 44 articles while the methods and the results section claim 42 articles were reviewed. The conclusions does not add important new knowledge to what is already known from the existing scientific literature.

Thank you very much, we appreciate this feedback. The following has been added to highlight the new knowledge generated in this review:
"By synthesising evidence across physical, psychological, emotional, and social health outcomes, rather than focusing on educational outcomes. It highlights the limited attention to children's psychological and emotional wellbeing, the role of stigma in targeted programmes, and the advantages of universal provision for promoting equity. It also identifies a critical need for more evidence from low- and middle-income countries."
(Page 33, Lines 741-746).

<u>Comments on paper</u>

How do school feeding programmes specifically alleviate child poverty? I see what you're trying to point to here; however, I think your point could be better made with an edit to this sentence. The literature on child poverty, food insecurity, and malnutrition is very nuanced, and I think it's important to be mindful of that here.

Thank you. An edit has been made as suggested. This now reads:

"School Feeding Programmes (SFP) are widely implemented to address child poverty, food insecurity, and malnutrition, yet evidence on their influence on children's health outcomes is limited."
(Page 2, Lines 28-30).

Please see comment in the abstract. While I can follow what you're trying to say here, I'm not sure that this sentence is entirely true as written.

Sentence edited as suggested. Sentence now reads as 'School feeding programmes (SFPs) are among the most widely implemented interventions intended to support child nutrition, improve food security, and reduce some of the immediate effects of poverty on children's wellbeing'
This sentence is unclear and requires rewording.

This sentence has been re-worded from: Moreover, in 2019, over 161 million children under five years of age were reported as having a nutritional deficiency globally from undernourishment.

To: Moreover, in 2019, over 161 million children under the age of five were affected by undernutrition globally [15]. Nutritional deficiencies associated with undernutrition have been linked to childhood wasting, stunting, infections, and cognitive and behavioural disorders.
(Page 4, Lines 105-108).

This is the first mention of these dimensions of health. Recommend defining health earlier in the introduction to include these dimensions (e.g. social, emotional physical and psychological)

Thank you for your comment. These health outcomes have been defined earlier in the introduction as recommended. (Page 3, Lines 87-89).

Provide a rationale for this year restriction: Studies published from 2009 were Included.

This has now been updated to 'This cut-off was selected to reflect recent developments in school feeding policies and global nutrition initiatives.'
(Page 7, Lines 169-170).

It may be more streamlined if you remove tables 1 and 2 and add them to table 3, so that the quality assessment is a colomn in the summary of the included papers.

Thank you for this suggestion. Tables 1 & 2 have been removed and added as an additional column in table 3 (now table 1).

Having these points as separate sentences makes the reading more challening. It may be more appropriate to separate some of the points using comas or semi-colons?

Thank you for pointing this out. This has now been changed. (Page 13, Lines 289-294).
Based on what data? It may be better to say that "Whilst targeted systems help to address food insecurity and child hunger..."

Sentence has been re-structured to 'Whilst targeted systems help to address food insecurity and child hunger' as suggested. (Page 16, Lines 322-323).

I appreciate the bold headings throughout the results; however, it would be helpful to state these headings here. "Findings are discussed under the following themes/headings (list them) and are described in more detail below

Thank you for your comment. This has now been changed to 'Findings are discussed under the following themes/headings: *Physical Health: BMI and Body Weight Outcomes, Nutritional Intake, Psychological and Emotional Health Outcomes, Social Health Outcomes, Reducing Food Insecurity and Child Hunger, Factors Influencing Uptake and Wider Impacts*. These are described in more detail below.' (Page 16, Lines 325-328).

Please revise this sentence to be more clear. You had separated physical, social, emotional, and psychological, but seem to be stating that all are physical?

Sentence has been revised from 'From a health perspective, SFP positively influenced children's physical health in terms of their social, emotional, and psychological well-being' to: 'From a health perspective, SFP positively influenced children's physical health, as well as their social, emotional, and psychological well-being.'
(Page 30, Lines 670-672).

What might be reasons for this difference? Does this highlight inter-country inequities? It would strengthen your discussion to add a piece on this.

Sentence has been revised and now reads: 'making comparisons difficult, these differences may reflect broader inter-country inequities in funding, infrastructure, and policy prioritisation of school feeding programmes.'
(Page 31, Lines 686-688).

Please state what some of these limitations are.

Limitations have now been added: 'Many rely on self-reported data, use small or context-specific samples, or apply inconsistent measures of emotional and psychological health. These limitations make it difficult to draw firm conclusions or compare findings across studies. As such, more robust and longitudinal research is needed to evaluate the impact of SFPs on children's psychological and emotional outcomes.'
(Page 31, Lines 674-678).

Can you speak to any inter-country differences in results and the implications of these?

A small paragraph has been added to implications for practice to discuss inter-country differences as advised. (Page 31, Lines 686-688).

VERSION 2 - REVIEW

**Reviewer**	**2**
**Name**	**Dokova, Klara**
**Affiliation**	**Medical University of Varna, Varna, Bulgaria**
**Date**	**30-Aug-2025**
**COI**	

The manuscript is substantially improved. The topic is very important and interesting.

My recommendations related to the abstract are taken inconsideration – the abstract is adequately revised.
The research question is revised and clarified.

There are still some small technical points that I would like make:

1. In the methods section under the subtitle :Quality assessment" there is a sentence: "Each section is assessed based on five criteria for methodological quality, offering three responses:…"
   It does not become clear why the authors write "each section" – is that each section of each paper that they review, or the whole paper?

2. The quality assessment paragraphs states: "Details of each study's quality rating and design are provided in Table 1."

The heading raw of "*Table 1:* Characteristics of the included studies" is as follow:

**Author**	**Country**	**Provision Type**	**Participant School year**	**Design**

It seems that "Quality" is missing.

3. Related to Table 1, it will be nice if the rows are numbered. There are two references in Table 1 - Carlisle et al. [74] and Jessiman et al [62] that mention "London" in the column named "Country"?
4. In the results section under the subtitle "***Nutritional Intake"*** – 10 papers are mentioned, however the cited list includes of nine references: [22,45, 44, 55, 53, 67, 46, 54, 49], probably No 60 is misisng? [60] Parnham JC, Laverty AA, Majeed A, Vamos EP. Half of children entitled to free school meals did not have access to the scheme during COVID-19 lockdown in the UK. Public Health. 2020;187:161-164. doi:10.1016/j.puhe.2020.09.006

5. The references are further mixed as under the heading "***Psychological and Emotional Health Outcomes***" **one sentence mentions*:* "**Taylor et al. [60]…." – reference 60 is [60] Parnham JC, Laverty AA, Majeed A, Vamos EP. Half of children entitled to free school meals did not have access to the scheme during COVID-19 lockdown in the UK. Public Health. 2020;187:161-164. doi:10.1016/j.puhe.2020.09.006

6. ***In the conclusion there is one sentence that seems unfinished: "***By synthesising evidence across physical, psychological, emotional, and social health outcomes, rather than focusing on educational outcomes."

VERSION 2 - AUTHOR RESPONSE

<u>Responses to Reviewer 1</u>

How do school feeding programmes specifically alleviate child poverty? I see what you're trying to point to here; however, I think your point could be better made with an edit to this sentence. The literature on child poverty, food insecurity, and malnutrition is very nuanced, and I think it's important to be mindful of that here.

Thank you. An edit has been made as suggested. This now reads:

"School Feeding Programmes (SFP) are widely implemented to address child poverty, food insecurity, and malnutrition, yet evidence on their influence on children's health outcomes is limited."
(Page 2, Lines 28-30).

Please see comment in the abstract. While I can follow what you're trying to say here, I'm not sure that this sentence is entirely true as written.

This sentence has been edited as suggested. Sentence now reads as 'School feeding programmes (SFPs) are among the most widely implemented interventions intended to support child nutrition, improve food security, and reduce some of the immediate effects of poverty on children's wellbeing'

This sentence is unclear and requires rewording.

This sentence has been re-worded from: Moreover, in 2019, over 161 million children under five years of age were reported as having a nutritional deficiency globally from undernourishment.

To: Moreover, in 2019, over 161 million children under the age of five were affected by undernutrition globally [15]. Nutritional deficiencies associated with undernutrition have been linked to childhood wasting, stunting, infections, and cognitive and behavioural disorders.
(Page 4, Lines 105-108).

This is the first mention of these dimensions of health. Recommend defining health earlier in the introduction to include these dimensions (e.g. social, emotional physical and psychological)

Thank you for your comment. These health outcomes have been defined earlier in the introduction as recommended. (Page 3, Lines 87-89).

Provide a rationale for this year restriction: Studies published from 2009 were

Included.

Thank you. This has now been updated to ‘This cut-off was selected to reflect recent developments in school feeding policies and global nutrition initiatives.’
(Page 7, Lines 169-170).

It may be more streamlined if you remove tables 1 and 2 and add them to table 3, so that the quality assessment is a colomn in the summary of the included papers.

Thank you for this suggestion. Tables 1 & 2 have been removed and added as an additional column in table 3 (now table 1).

Having these points as separate sentences makes the reading more challening. It may be more appropriate to separate some of the points using comas or semi-colons?

Thank you for pointing this out. This has now been changed. (Page 13, Lines 289-294).

Based on what data? It may be better to say that "Whilst targeted systems help to address food insecurity and child hunger..."

Sentence has been re-structured to ‘Whilst targeted systems help to address food insecurity and child hunger’ as suggested. (Page 16, Lines 322-323).

I appreciate the bold headings throughout the results; however, it would be helpful to state these headings here. "Findings are discussed under the following themes/headings (list them) and are described in more detail below

Thank you for your comment. This has now been changed to 'Findings are discussed under the following themes/headings: *Physical Health: BMI and Body Weight Outcomes, Nutritional Intake, Psychological and Emotional Health Outcomes, Social Health Outcomes, Reducing Food Insecurity and Child Hunger, Factors Influencing Uptake and Wider Impacts.* These are described in more detail below.'
(Page 16, Lines 325-328).

Please revise this sentence to be more clear. You had separated physical, social, emotional, and psychological, but seem to be stating that all are physical?

Thank you. This sentence has been revised from 'From a health perspective, SFP positively influenced children's physical health in terms of their social, emotional, and psychological well-being' to: 'From a health perspective, SFP positively influenced children's physical health, as well as their social, emotional, and psychological well-being.'
(Page 30, Lines 670-672).

What might be reasons for this difference? Does this highlight inter-country inequities? It would strengthen your discussion to add a piece on this.

Sentence has been revised and now reads: 'making comparisons difficult, these differences may reflect broader inter-country inequities in funding, infrastructure, and policy prioritisation of school feeding programmes.'
(Page 31, Lines 686-688).

Please state what some of these limitations are.

Limitations have now been added: 'Many rely on self-reported data, use small or context-specific samples, or apply inconsistent measures of emotional and psychological health. These limitations make it difficult to draw firm conclusions or compare findings across studies. As such, more robust and longitudinal research is needed to evaluate the impact of SFPs on children's psychological and emotional outcomes.'
(Page 31, Lines 674-678).

Can you speak to any inter-country differences in results and the implications of these?

A small paragraph has been added to implications for practice to discuss inter-country differences as advised. (Page 31, Lines 686-688).

Response to Reviewer Two

In the methods section under the subtitle :Quality assessment" there is a sentence: "Each section is assessed based on five criteria for methodological quality, offering three responses:…"

It does not become clear why the authors write "each section" – is that each section of each paper that they review, or the whole paper?

Thank you for pointing this out. This sentence has now been amended to clarify. (Page 9, line 233).

The quality assessment paragraphs states: "Details of each study's quality rating and design are provided in Table 1."

Thank you for this observation. The table has now been updated to include quality.

Related to Table 1, it will be nice if the rows are numbered. There are two references in Table 1 - Carlisle et al. [74] and Jessiman et al [62] that mention "London" in the column named "Country"?

This has now been corrected to 'England.'

In the results section under the subtitle "*Nutritional Intake*" – 10 papers are mentioned, however the cited list includes of nine references: [22,45, 44, 55, 53, 67, 46, 54, 49], probably No 60 is misisng? [60] Parnham JC, Laverty AA, Majeed A, Vamos EP. Half of children entitled to free school meals did not have access to the scheme during COVID-19 lockdown in the UK. Public Health. 2020;187:161-164. doi:10.1016/j.puhe.2020.09.006

Thank you. This has now been amended.

The references are further mixed as under the heading "*Psychological and Emotional Health Outcomes*" one sentence mentions*:* "Taylor et al. [60]…." – reference 60 is [60] Parnham JC, Laverty AA, Majeed A, Vamos EP. Half of children entitled to free school meals did not have access to the scheme during COVID-19 lockdown in the UK.

Public Health. 2020;187:161-164. doi:10.1016/j.puhe.2020.09.006

Thank you for noting this. The reference has been changed to the correct number 'Traylor et al., [64]. (Page 21, line 474).

In the conclusion there is one sentence that seems unfinished: **"By synthesising evidence across physical, psychological, emotional, and social health outcomes, rather than focusing on educational outcomes."**

Thank you for pointing this out. The grammar of the sentence has been changed so the sentence is finished. (Page 32, line 748)
